# *Capparis spinosa* L. in A Systematic Review: A Xerophilous Species of Multi Values and Promising Potentialities for Agrosystems under the Threat of Global Warming

**DOI:** 10.3389/fpls.2017.01845

**Published:** 2017-10-25

**Authors:** Stephanie Chedraoui, Alain Abi-Rizk, Marc El-Beyrouthy, Lamis Chalak, Naim Ouaini, Loïc Rajjou

**Affiliations:** ^1^IJPB, Institut Jean-Pierre Bourgin (INRA, AgroParisTech, CNRS, Université Paris-Saclay), Saclay Plant Sciences (SPS)-RD10, Versailles, France; ^2^Faculty of Agricultural and Food Science, Holy Spirit University of Kaslik, Jounieh, Lebanon; ^3^Faculty of Agricultural Sciences, Lebanese University, Beirut, Lebanon

**Keywords:** *Capparis spinosa* L., drought tolerance, cultivation, agronomy, taxonomy, genetic analysis, phytochemical, traditional use

## Abstract

Caper (*Capparis spinosa* L.) is a xerophytic shrub with a remarkable adaptability to harsh environments. This plant species is of great interest for its medicinal/pharmacological properties and its culinary uses. Its phytochemical importance relies on many bioactive components present in different organs and its cultivation can be of considerable economic value. Moreover, taxonomic identification of *C. spinosa* L. has been difficult due to its wide heterogeneity, and many authors fell into confusion due to the scarcity of genetic studies. The present review summarizes information concerning *C. spinosa* L. including agronomic performance, botanical description, taxonomical approaches, traditional pharmacological uses, phytochemical evaluation and genetic studies. This knowledge represents an important tool for further research studies and agronomic development on this indigenous species with respect to the emerging climatic change in the Eastern Mediterranean countries. Indeed, this world region is particularly under the threat of global warming and it appears necessary to rethink agricultural systems to adapt them to current and futures challenging environmental conditions. *Capparis spinosa* L. could be a part of this approach. So, this review presents a state of the art considering caper as a potential interesting crop under arid or semi-arid regions (such as Eastern Mediterranean countries) within the climate change context. The aim is to raise awareness in the scientific community (geneticists, physiologists, ecophysiologists, agronomists, …) about the caper strengths and interest to the development of this shrub as a crop.

## Introduction

In a world likely to be challenged by the threat of global warming, it is expected to observe negative effects on growth and reproductive success of plants. The Mediterranean region has been pointed out as a climate change hot spot by the IPCC (Intergovernmental Panel on Climate Change; http://www.ipcc.ch; Pachauri et al., 2014). Evidences of substantial impact on agricultural production are already occurring. High temperatures, heat waves and drought stress leading to loss in plant productivity might result in an inability to ensure global food security (Bita and Gerats, 2013; Ray et al., 2015). For instance, wheat crop yields fell by 25–35% with a 3–4°C rise in temperature in the Middle East (Ortiz et al., 2008). Various molecular, cellular, physiological and morphological damages have been observed under elevated temperatures, leading to a decrease in plant growth (Vollenweider and Günthardt-Goerg, 2005; Hatfield and Prueger, 2015; Ohama et al., 2017). In many cases, aridity, excessive heat and elevated CO_2_ cause modifications in respiration and photosynthesis, leading to a reduced plant life cycle and a loss in plant productivity (Prasad et al., 2008; Yamori et al., 2014; Xu et al., 2015).

Nevertheless, the introduction of stress tolerant crops and cultivars in agrosystems is not a rapid process due to the long delays between laboratory research and validation of field trials. Such crops might constitute an efficient way to cope with the foreseeable nutritional needs and to promote a sustainable agriculture (Thiry et al., 2016). In this context, this review gives attention to a xerophilous crop, well adapted to drought and of promising potentialities namely caper (*Capparis spinosa* L.). Caper is a Mediterranean shrub known for its edible flower buds and fruits pickled in salt and vinegar. This species possesses strong characteristics of adaptation to the regions displaying fluctuating climate and is a candidate for being domesticated to maintain and promote agriculture in regions subject to extreme climate change and affected by hyper-aridity. The advantages of using such xerophilous species include their moderate water requirements, a high potential for genetic improvement, local knowledge and know-how on this plant material and an existing global trade chain for the use of plant products. Perennial plantations of caper could contribute to preserve water in the soil for a longer period of time and can help to maintain sustainable agroecosystems. Such shrubs protect the soil from sunlight, limiting high soil temperatures and thus regulating the microclimate. By comparison with other desert plants, caper has a high water use efficiency (WUE) and a remarkable ability to search and absorb water from its environment (particularly in soil depths) thanks to an extensive root system and a very high root/stem ratio (Zuo et al., 2012; Gan et al., 2013). This root system is very effective for water retention during scattered rainfall events, providing suitable conditions for soil fauna and microbiota development. Caper plantations can be associated with annual plants (e.g., vegetables, grassland plants, medicinal herbs) to improve biodiversity and provide multiple benefits (Solowey, 2010). In addition, it has a considerable economic importance through the uses of its roots, buds and fruits in many food and pharmaceutical industries (Sher and Alyemeni, 2010). *C. spinosa* L. has an aesthetic blossom and a sweet-scented flower, thus it is used as an ornamental plant for gardens and walls as well for terraces exposed to sun. It requires no watering and can be grown in poor soils or even stones (Gan et al., 2013). At the agronomic level, this species has led to great financial returns from its cultivation due to its resistance to environmental stresses and its enormous ethnobotanical and pharmaceutical importance, as well as its content in bioactive agents having high nutritional value and great efficacy in the manufacture of medicines and cosmetics. Nevertheless, in the East Mediterranean countries, *C. spinosa* has not yet been sufficiently exploited due to the scarcity of buds consumption at the local level (Chalak et al., 2007).

Few studies have reviewed *C. spinosa* focusing on the plant nutritional quality, food and medicinal uses, phytochemistry, ethnopharmacology, biological activities and cultivation (Rivera et al., 2003; Sozzi and Vicente, 2006; Tlili et al., 2011a; Gull et al., 2015; Nabavi et al., 2016). *C. spinosa* displays huge agro-based potentialities and a highly demand for exploitation due to a diversified international market. Today, it seems necessary to focus on the possibility of selection and improvement of this specie and to develop more intensive research to promote this crop, especially in the east Mediterranean countries. Actually, the impacts of climate change are already being felt by the Arab region (UNEP/ROWA, 2015). Rural communities of this region are the first to be vulnerable to such changes. This could be overcome by exploiting and enlarging the cultivation of existing well adapted flora and by the development of crops highly tolerant to drought and heat stress. The awareness in agro-biodiversity for selecting the development of *C. spinosa* as a multipurpose crop that proved to have better resistance to drought and harsh environmental conditions is a significant need to alleviate climate change effects in agro-ecosystems of East Mediterranean region.

## Origin and distribution

### Origin and discovery

The *Capparis spinosa* Linnaeus (1753: 503) group belongs to the *Capparis* genus *sect. Capparis* created and described by Carolus Linnaeus in his book “species Plantarum” (Inocencio et al., 2006). The genus *Capparis* belongs to the Capparidaceae family, closely related to Brassicaceae (Hall et al., 2002; Inocencio et al., 2006) and includes 350 species of tropical or subtropical origin, many of them distributed in the Mediterranean regions (Fici, 2001; Inocencio et al., 2006). *C. spinosa* was described as a hybrid between *C. orientalis and C. sicula* (Rivera et al., 2002). Caper is the English common name of this genus and it is also known by different names, e.g., Kabbar (Arab), câprier (French), and Alcaparro (Spain) (Zohary, 1960; Heywood, 1964; Jacobs, 1965; Inocencio et al., 2006; Saadaoui et al., 2007). Archaeological discoveries from an Old-World Paleolithic site in Egypt suggested *Capparis* spp. consumption since 17,000 years ago (Hillman, 1989; Hansen, 1991). Seed of *C. spinosa* L. were found at Tell es-Sawwan (Iraq, 5800 BC) and in the Yanghai Tombs of Turpan District in Xingjiang-China (2800 B.C.) (Renfrew, 1973; Jiang et al., 2007). The plant was used since ancient Greeks, Hebrews and Romans at Tell es Sweyhat-Syria. Pickled Capers consumption dates back to the Bronze Age. (Van and Bakker-Heeres, 1988; Sozzi, 2001). In the Middle East, Zohary regarded *Capparis* as a native flora distributed in Africa and south-western Asia (Zohary, 1960), whereas Jacobs suggested that the Malaysian and Australian *C. spinosa* were introduced by humans (Jacobs, 1960).

### Geographic distribution

**Figure 1:**
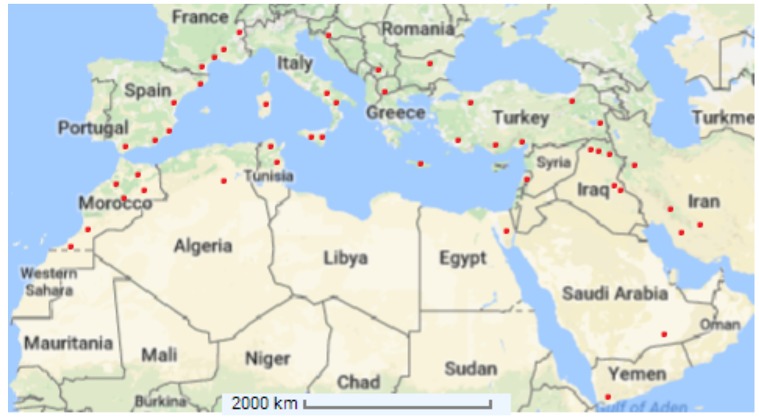
Distribution of *Capparis spinosa* L. (•) over the Mediterranean Basin (Adapted from Inocencio et al., 2006).

*Capparis spinosa* grows naturally from the Atlantic coast of the Canary Island and Morocco to the Black Sea, in Crimea and Armenia, and to the east side of the Caspian Sea and Iran (Alkire, 1998; Inocencio et al., 2002). It is spread in North Africa, Europe, West Asia, Afghanistan, and Australia (Willis, 1988). This plant might have aroused in the tropics, and then extended to the Mediterranean basin and Central Asia (Zohary, 1960). Different subspecies and varieties have specific geographic distributions. *C. spinosa* subsp. *spinosa* is distributed in Southern Europe, northern Africa including Sahara, Arabic peninsula, and Middle East to China. *C. spinosa* subsp. *rupestris* is widespread in France, Italy, Spain, Slovenia, Malta, Croatia and Albania and also reported in Turkey, Greece, Algeria, Libya and Tunisia (Inocencio et al., 2006; Fici, 2015; see Figure 1). The Mediterranean regions might be harshly affected by global warming, leading to extensive effects on agroecosystems and crop production. A particular attention should be paid to plants adapted to arid conditions for being used in agricultural systems under the current climate change scenario.

## Botanical and taxonomic studies

### Botanical description

Species belonging to the genus *Capparis* have plesiomorphic features (Fici, 2001). Some available literature treated the botanical description of *Capparis spinosa* and reported the polymorphic aspects of this species and the high degree of heterogeneity in its morphological characters (Post, 1932; Zohary, 1960; Mouterde, 1968; Higton and Akeroyd, 1991; Legua et al., 2013). The latter being slightly zygomorphic, abaxial sepal not galeate or slightly galeate with numerous stamens (Inocencio et al., 2006).

The species *C. spinosa* is a winter-deciduous perennial shrub. It is erect, precumbent or pendulous with branches being unramified or multi-ramified, green, red or yellow, attending 4 m long. Twigs are tortuous or straight, with or without simple hairs. Stipules are somewhat curved, straight, setaceous or spreading, antrorse or retrorse, orange, yellow or green, reaching 6 mm long. Leaf stipules may be formed into spines, granting it the name “spinosa.” Leaves are rounded, or ovate, lanceloate or oblong, ellipticial or obordate with an obtuse, tapering, acute or cordate base and an acute, rounded, obcordate, truncate or obtuse apex. Leaf veins are prominent or not. Leaf texture can be glabrous, pubescent and very dense. Petiole is grooved or entire, 0–2 cm. Flowers are solitary, somewhat zygomorphic mainly noctoflorous. Four white or white-pinkish petals, oblong, obovate or rounded-ovate. Stamens are numerous with filaments up to 5 cm. Gynophore is 3–6 mm long. Fruit is ellipsoidal, obovate or oblong. Seeds are numerous and reddish-brown (Inocencio et al., 2006; Fici, 2014). Additionally, physiological capacities enabling adaptation of *C. spinosa* to drought conditions were ascertained. The plant might change its leaf, stem and root structure when facing dry areas. The xylem and fibro-vascular systems increase and the transit region between the root and stem enlarges in order to boost water absorption and storage capacity (Gan et al., 2013).

### Taxonomic description

Taxonomic studies based on the shrub leaf and flower phenotypes revealed a complex variation pattern within variants of *C. spinosa* on different landmasses (Zohary, 1960). Consequently, this made the identification of the *C. spinosa* group very complicated in the Mediterranean region. Many taxa at various ranks of classification have been described in the Middle East (Zohary, 1960; Maire, 1965; Inocencio et al., 2006; Danin, 2010).

A previous study indicated that *C. spinosa* is morphologically related to *C. sicula* Duhamel as well to *C. orientalis* Duhamel and overlaps with the latter (Inocencio et al., 2005). Recently a taxonomic revision has been conducted by Fici (2014, 2015) on the *C. spinosa* group widespread from the Mediterranean to central Asia. *C. spinosa* is recognized as a single species and is represented by four subspecies (i.e., *C. spinosa* subsp. *spinosa; C. spinosa* subsp. *rupestris*; *C. spinosa* subsp. *cordifolia*; *C. spinosa* subsp. *himalayensis). C. spinosa* subsp. *spinosa* is widely distributed eastwards from the Mediterranean to China and Nepal, showing inherited traits and great level of heterogeneity. Within this subspecies, some varieties are identified, namely var. *herbacea* and var. *atlantica. C. spinosa* subsp. *rupestris* is less diversified and more similar to the tropical lineage. Two varieties were also recognized, var. *ovate* and var. *myrtifolia*.

A more recent study investigated *C. spinosa* forms distributed in the Paleotropis, Australia and in a few tropical areas of northern-eastern Africa and southern Asia. Two original nomenclatures are proposed, i.e., C. *spinosa* subsp. *cordifolia* comb. et stat. nov. and *C. spinosa* subsp. *himalayensis* stat. nov. (Fici, 2015).

## Genetic diversity

*Capparis spinosa* shows considerable morphological variation due to various factors such as phenotypic plasticity, eco-geographical differentiation, topographical modifications, and hybridization processes promoting the presence of intermediate phenotypes. This high variability suggests chaotic complex structure within wild forms of *C. spinosa*. The pure morphological approaches based solely on qualitative and quantitative vegetative characters have led to much confusion in the taxonomy of *C. spinosa*, with misidentification of the taxon and erroneous classification of the different varieties. Therefore, research that deals with molecular data has greatly complemented morphological classifications and has helped in revealing the phylogenetic relationships, with different eco/biotypes and the evolutionary trends of this species. At present, a few number of studies reported molecular data in studying the taxonomy of *C. spinosa* and its genetic profile (Table 1).

**Table 1:** Genetic data available for the *Capparis spinosa* L. group in the Mediterranean and Near East.

**References**	**Geographic origin**	**Markers**	**Results**
Wang et al., 2016	China	cpDNA	Genetic differentiation by vicariance.
Liu et al., 2015	China	ISSR	Genetic clustering linked to geographic factors.
Al-Safadi et al., 2014	Syria	IRAP & ISSR	Genetic distinction between *Capparis* sp. with possible hybrid origin.
Silvestre et al., 2014	Sicily-Itlay	ISSR	Genetic discrimination between two subspecies.
Ozbek and Kara, 2013	Turkey	RAPD	Genetic differentiation of five varieties.
Bhoyar et al., 2012	India	RAPD and ISSR	Geographical distribution and genetic variation are correlated.
Nosrati et al., 2012	Azerbaijan Iran	RAPD	Genetic variation in small population is lower than that in large population.
Saifi et al., 2011	Morocco	ISSR	Genetic distance partially related to geographical distances.
Moubasher et al., 2011	Egypt	ISSR	Three varieties of *C. spinosa*. var. inermis suggested as independent species.
Inocencio et al., 2005	Spain, Morocco, Syria	AFLP	*C. spinosa* is a hybrid resulting from *C. orientalis* with introgression from *C. sicula*.
Khouildi et al., 2000	Tunisia, Central Italy	RAPD	Genetic variation is related to environmental factors rather than geographical distances.

Based on Amplified Fragment Length Polymorphism (AFLP) a low genetic distance was revealed among *Capparis* sp. (i.e., *C. spinosa, C. orientalis, C. sicula, C. aegyptia*, and *C. ovata*) from Spain, Morocco and Syria (Inocencio et al., 2005). About 50% of polymorphic frequency was revealed between *C. orientalis, C. spinosa* and *C. sicula* and a low consistency of *C. spinosa*, with 2% unique bands was marked. A possible hybrid origin of *C. spinosa* was suggested, comprising cultivars from different lineages of *C. orientalis* with some introgression from *C. sicula* thus a greater genetic influence from *C. orientalis* due to the unfrequented presence of *C. sicula* in the studied area (Balearic Islands) (Inocencio et al., 2005).

In Egypt, the taxonomic identity among and within species of the genus *Capparis* using Random Amplified Polymorphism DNA (RAPD) was conducted by Moubasher et al. (2011). Eight polymorphic RAPD markers were generated. A considerable genetic variation was identified and revealed the presence of three varieties of *C. spinosa*: var. *spinosa*, var. *canescens*, var. *deserti* and one inermis type. *C. spinosa* var. *inermis* was closer genetically to *C. sinaica* than to *C. spinosa* var. *spinosa, C. spinosa* var. *canescens*, and *C. spinosa* var. *deserti*. Thus *C. spinosa* var. *inermis* was suggested to be treated as independent species.

The genetic assessment of Moroccan capers by Inter Simple Sequence Repeat (ISSR) revealed 98.89% distinct profiles based on the geographic origin and indicated remarkable phenotypic plasticity linked to the ecological area and environment (Saifi et al., 2011). This might be explained by a low level of gene flow due to the fragmentation of habitats of these populations that leads to accumulate significant genetic differences (Inocencio et al., 2005). The genetic study of Azerbi and Iranian Capers using RAPD markers indicated no correlation between genetic variation and geographical distances among populations (Nosrati et al., 2012). Nevertheless, the same study revealed that those genetic distances were significantly lower in small populations than those in large populations with a percentage of polymorphic RAPDs bands ranging from 42 to 67% in small-sized populations and from 70 to 81% in large-sized populations. Moreover, 32.83% of total genetic variation was shared among populations while 67.17% restricted to within-populations, indicating an important fragmentation of habitats in this region.

Bhoyar et al. (2012) analyzed the genetic variability of *C. spinosa* populations growing in the trans-Himalayan region in India for adaptation to high altitude, by using both RAPDs and ISSRs markers. ISSRs were more efficient for detecting polymorphism in caper where microsatellites containing the repeated di-nucleotides (AG)n, (AC)n, (TG)n, (GA)n, and trinucleotides (ACC)n, and (GGC)n were frequent in caper. Geographical distribution and genetic variation were correlated, which can be explained as a sign of a longstanding pattern of restricted gene flow (Bhoyar et al., 2012).

In Turkey, Ozbek and Kara (2013) differentiated five varieties: *C. spinosa* var. *spinosa*, var. a*egyptia* and var. *canescens*, and *Capparis ovate* Desf. var. *palaestina*, and var. *herbacea*. Ten RAPD primers produced 98 loci, 73 of which were polymorphic with 87.42% total genetic variation. Hypothesis of the effect of population size on genetic diversity was confirmed as well as the relation between eco-geographical factors and genetic diversity affecting the number of effective alleles.

Silvestre et al. (2014) investigated capers growing in Sicily and the surrounding islets of Lampedusa, Pantelleria and Salina using ISSR markers. The results strongly supported morphological analysis and discriminated between the two subspecies *spinosa* and *rupestris*, indicating that genetic diversity can be related to environmental conditions rather than geographical distances. On the other hand, intermediate phenotypes showed hybridization between the two taxa for almost 80% in contact zones while cultivated biotypes presented genetic affinity to subsp. *rupestris*.

A recent study conducted in Syria correlated the morphological traits to the genetic differentiation and to the geographical distribution of *Capparis* species, using Inter Retro-transposon Amplified Polymorphism (IRAP), ISSR and combined data of IRAP+ISSR. The percentages of polymorphism recorded were 71, 82, and 75%, respectively for the three techniques. A clear separation was revealed among *C. spinosa* L., *C. aegyptia* Lam, and *C. sicula* Duh. Nevertheless, two samples could not be identified and were found at an intermediate position between *C. sicula* and *C. spinosa* indicating a possible hybrid origin between these two species (Al-Safadi et al., 2014).

The first genetic analysis of Chinese *Capparis spinosa* populations revealed the classification of the three distinct groups geographically separated and showed high genetic diversity using ISSR markers (Liu et al., 2015). In Western Himalayas, Tianshan Mountains and adjacent desert regions, vicariance phenomenon was suggested to explain genetic clades of *C. spinosa* identified based on three chloroplast DNA (cpDNA) fragments (Wang et al., 2016).

## Cultivation and production

### Environmental conditions

*Capparis spinosa* L. is a species of arid and semi-arid climate zones and is well known as a highly drought tolerant plant. It is one of a few species that grow and flower in summer in arid regions. In the Mediterranean basin, it is free of competition for water with other species (Rhizopoulou et al., 1997; Rhizopoulou and Psaras, 2003). It requires a semi-arid climate with mean annual temperatures over 14°C and mean annual rainfall not less than 200 mm. It is adapted to xeric areas, therefore, it can bear up water stress without any manifestation, and resists strong winds and temperatures exceeding 40°C in dry Mediterranean summers (Sozzi and Vicente, 2006). Moreover, caper survives winters in the form of stump; yet, frost can be disturbing during its vegetative period. It is usually grown at low altitudes even though some plants were found even over 1,000 m above sea-level (Barbera, 1991; Chalak et al., 2007).

*C. spinosa* was described as both a rupicolous and a stenohydric plant (Rhizopoulou et al., 1997). Stenohydric plants have not developed dehydration avoidance to as a degree as in desiccation-tolerant organisms such as resurrection plants. Caper plant adapts to calcareous soils or moderate percentages of clay (González, 1973). It has an efficient root system associated with nitrogen fixing bacteria that allows the growth in soils with poor fertility (Andrade et al., 1997). It also tolerates salty, sandy, or rocky soils, with low amount of organic matter as in India (Ahmed, 1986; Kala and Mathur, 2002). It prefers saline and halophytic habitats (Al-Yemeni and Zayed, 1999). Caper is also wildly grown in wall joints and in antique monuments (Barbera, 1991; Chalak et al., 2007).

*C. spinosa* has low flammability thus might be used for cutting down wild forest fires which are Mediterranean climate characteristics (Neyisci, 1987). *C. spinosa* is utilized for landscaping, it reduces erosions along steep rocky slopes, highways, sands dunes or fragile semiarid ecosystems (Faran, 2014). *C. spinosa* is a promising species due to its potential use in agroforestry and its ability to protect land in Mediterranean countries (Sher et al., 2012).

### Ecophysiological aspects and adaptation traits

The xeromorphic features of *C. spinosa* have been highlighted in several studies (Rhizopoulou, 1990; Rhizopoulou and Psaras, 2003; Sakcali et al., 2008; Wang et al., 2016).

Anatomical adaptations to aridity include root, stem, leaf and flower features. As mentioned above, a major aspect that may explain the high resistance of wild *C. spinosa* to drought concerns its extremely deep root system (Özkahraman, 1997). Caper root system represents 62.5% of the total plant biomass after 4–5 months of growth (Sozzi, 2001; Gan et al., 2013). Roots also excrete acidic compounds that can perforate rocks and cracks to reach water resources (Oppenheimer, 1960). In addition, the xylem vessels in stems are extremely well developed in *C. spinosa*, leading to an efficient hydraulic conductivity (Psaras and Sofroniou, 1999; Levizou et al., 2004). It is worth noting that the thick cortical layers in tap and fibrous roots and a swollen transfer region are able to store water and protect fibro-vascular bundle against damage under drought conditions (Gan et al., 2013).

At the leaf level, thick, small and multi-layered mesophyll cells were also found in *C. spinosa*. The small leaf intercellular air space percentage of 15% and the thick terminal epidermal cell walls are characteristic traits of xerophytes. Moreover, the wax-like and water-repellent cutin covering the epidermis and the shapely trichomes help the growing of *C. spinosa* in arid areas (Li et al., 2007). The well-developed sclerenchymatic tissue and the differentiated palisade parenchyma allow to maintain the protection of *C. spinosa* leaves against irreversible damages during severe water stress (Stefanou and Manetas, 1997; Rotondi et al., 2003). Stomata are the main channels for transpiration and are widely and evenly distributed across both leaf surfaces and are able to stay opened a full day. The opening of the stomata promotes evapotranspiration and has a strong cooling effect on leaf temperature in desert environments. Stomata were also found on the adaxial and abaxial surfaces of the petals and vacuolated parenchyma cells with large intercellular space. The membrane fluidity is influenced by the presence of unsaturated fatty acids, identified as major components of lipids in petals (Rhizopoulou et al., 2006). Under stress conditions, unsaturated fatty acids contribute to maintain membrane fluidity and its physiological functions. These traits offer a competitive advantage to this species.

The growth period and blooming of *C. spinosa* can occur entirely during dry and hot summers in the Mediterranean. It has been reported that the blooming of this shrub is not affected by severe water deficit (Vardar and Ahmed, 1972; Sheikh, 1976; Rhizopoulou and Psaras, 2003). Furthermore, high solar irradiance is very efficiently used by *C. spinosa* without any symptoms of photoinhibition. This photosynthetic performance makes *C. spinosa* a suitable candidate for being grown in drought areas, while most plants have minimum growth rates (Levizou et al., 2004).

### Seed propagation

One gram of fruit contains between 150 and 160 seeds (Gorini, 1981). Seeds are obtained by fruit rubbing followed by washing and drying in the shade (Sozzi and Vicente, 2006). Seed germination is the method of propagation mostly adopted for caper plant. The germination performance of caper seeds is poor due to a high dormancy and a low longevity. Seed viability is about 2 years when kept at 4°C and low relative humidity. Sprouted seeds are obtained after 25–50 days (Barbera, 1991). This traditional technique strongly limited by a low germination rate has been used in Argentina (Sozzi and Chiesa, 1995), Armenia (Ziroyan, 1980), Cyprus (Orphanos, 1983), India (Singh et al., 1992), Italy (Barbera and Di Lorenzo, 1984), Spain (Lorente and Vicente, 1985; Pascual et al., 2003), and USA (Bond, 1990).

The poor caper seed propagation is due to the weak germination capacity and to the hard coat of the seeds; therefore, the tough structure of the seed and the mucilage developing when placed in contact with water could limit the diffusion of oxygen to the embryo (Barbera, 1991; Bahrani et al., 2008). Indeed, the seed vigor (including speed and rate of germination) is affected by the maturity of the seeds, the fruit position and weight (Pascual et al., 2003). Different treatments are requested to overcome the prevailing dormancy to improve the germination (Sozzi and Chiesa, 1995). Among them, mechanical scarification (sand paper, ultrasound etc.), cold stratification, soaking in concentrated sulfuric acid (H_2_SO_4_), 0.2% KNO_3_, gibberellins (GA_4+7_ and GA_3_) and manipulation of the environmental conditions (light/dark, temperature) were efficient to promote caper seed germination.

### Asexual propagation

Use of stem cutting for propagation pays the serious rooting problems but has the advantages of avoiding high variability in terms of production and stability of quality traits. Vegetative propagation of caper allows to obtain numerous individuals from a limited number of plants. Stem cuttings can be obtained from hardwood, semi-hardwood or softwood (herbaceous) segments (Güleryüz et al., 2009). Hardwood cuttings vary in length from 1 to 50 cm and from 1 to 2.5 cm in diameter. Stems can be collected on February and March, treated with fungicides (e.g., captan or captafol) and then stratified outdoors or at 3–4°C and finally covered with sand or plastic (Lorente and Vicente, 1985). Semi-hardwood cuttings can be collected and planted on August and September, but low survival rates (under 30%) have been observed (Barbera, 1991). Softwood cuttings increase rooting percentage; they are collected and prepared on April (germination period) with basal or subterminal cuttings more successful than the terminal ones. Stem cuttings are planted under a mist system with heat that is believed to have a positive effect on rooting as well as dipping the cutting basal into auxin solution (1,500–3,000 mg/L) (Pilone, 1990). Hardwood cuttings do not seem to be influenced by hormonal treatments, whereas softwood cuttings gave 83% rooting percentages when treated with α-naphtaleneacetic acid (NNA) (Lorente and Vicente, 1985).

Propagation by grafting is a less adopted method for caper; however, it was carried out in Spain with acceptable results using bark grafting in planting (60% rooting) (Barbera, 1991) and could offer very interesting perspectives to develop caper hybrids (Zhou and Liu, 2015). *In vitro* propagation was successful from nodal shoot segments. Rodriguez et al. (1990) showed that 6-benzylaminopurine enhanced clusters proliferation when combined with indoleacetic acid and GA_3_. Gamma irradiation stimulated growth of shoots up to 200% and increased shoot rooting percentage from 75 to 100% according to Al-Safadi and Elias (2011). The *in vitro* micropropagation of *C. spinosa* was reported in several countries (Salem et al., 2001; Chalak et al., 2003; Caglar et al., 2005; Musallam et al., 2010; Carra et al., 2011, 2012). Chalak and Elbitar (2006) described a protocol for the micropropagation of a Lebanese morphotype (*C. spinosa* subsp. *rupestris*) using single nodal cuttings. High rates of shootlets rooting response (92%) was obtained after 4 h pulse treatment period in darkness with auxin, followed by culture on solid half strength Murashige and Skoog basal medium. Development of a tissue culture system is a promising approach to identify high-yielding lines. Micropropagation protocols for caper could be useful and efficient in producing desirable seedlings for transplanting.

### Cultivation, practices, and productivity

Caper plant phenology was reported using the BBCH scale (Biologische Bundesanstalt, Bundessortenamt, and CHemical industry) describing nine principal growth stages (Legua et al., 2013). The main traits of interest for cultivated caper bush are: high productivity, long stems, short internodes and high node fertility, dark green spherical flower buds with close non-pubescent bracts and late opening, oval fruit with light green pericarp and few seeds, absence of stipular spines, easy stalk separation to simplify harvest and postharvest operations, capacity for asexual reproduction and resistance to biotic and abiotic stresses (Barbera, 1991).

Caper is a spontaneously growing plant, though it is cultivated in several Mediterranean countries. It has already developed traits to survive new climate conditions. Therefore, its cultivation can help in adapting agricultural management to climate constraints in most Mediterranean regions (Howden et al., 2007).

*C. spinosa* is known as an economic plant in Australia and tends to spread in Latin America. The economic importance of caper has led to an increase in yield and production level. Specialized cultivation of caper started around 1970 in Spain and Italy, with a maximum of about 4,000 and 1,000 ha in cultivation, respectively in the 1990s. World caper production is estimated around 15–20,000 tons/year and the global trade concerns about 60 countries. Actually, Morocco and Turkey are the leading world producers and exporters (Infantino et al., 2007). Cultivation of caper is recorded in Spain, Italy and France, especially the Mediterranean island of Pantelleria, the Aeolian island of Salina and Sicily, where several local cultivars and ethnovarieties are known (Inocencio et al., 2006).

The most important Spanish cultivars (biotypes) are “Común” or “del País” and “Mallorquina” (Lorente and Vicente, 1985). Italian commercial biotypes are “Nocellara” (a cultivar within *C. orientalis*), and “Nocella.” Other Italian biotypes are “Ciavulara,” “Testa di lucertola,” “Spinoso of Pantelleria” and “Spinoso of Salina” (a cultivar within *C. sicula* subsp. *sicula*) (Barbera, 1991). “Redona,” “Roses,” “De las Muradas,” “FiguesSeques,” and “Peluda” are cultivated in a lower amount in the Balearic Islands: (Rivera et al., 1999). Nevertheless, caper cultivation is mostly restricted to *C. spinosa* but also the commercial product known as “Capers” is actually being obtained from the cultivated *C. spinosa, C. orientalis* and *C. sicula*, in addition to intermediate biotypes having an identical genetic constitution (Inocencio et al., 2005).

Caper bush is cultivated mostly in non-irrigated lands. Despite its ability to grow in drought conditions, irrigation is especially important during the first year when the caper bush is highly sensitive to water stress (Sozzi and Vicente, 2006). Moldboard plowing and harrowing are usual practices prior to caper cultivation (Lorente and Vicente, 1985).

Nursery plants, propagated as seedlings or rooted cuttings, are maintained in nursery row during the dormant season. Transplanting, either bare-root or containerized, takes place after the last frosts and is carried out by hand (Sozzi and Vicente, 2006).

Square/rectangle and hedgerow planting designs are used. Spacing is determined according to the fertility of the soil, the resistance of the biotype, the equipment to be used and the irrigation method employed. Bush spacing of 2.5 × 2.5 m, or 2.5 × 2 m, 3 × 3 m, 4 × 4 or 5 × 5 m are satisfactory (Barbera and Di Lorenzo, 1984; Bounous and Barone, 1989). Caper bush cultivation can also be associated with vine (as in Pantelleria, Italy), olives (as in Salina, Italy) or almonds (as in south Spain) (Barbera, 1991).

Harvest is the heaviest operation of Caper production. It may represent 2/3 of the total labor as it is done manually. Harvest is difficult and time-consuming due to the dropping branches, the presence of stipular spines in some biotypes, the small diameter of flower buds and the high temperatures and solar radiation during summer under Mediterranean climate. Yields of flower buds increase with age, from 1 to 9 kg/plant/year. A maximum yield is expected in the 4th year; however, caper bush yields are highly variable depending on the age, growing environment, cultural practices and biotype.

### Pests and diseases

*Capparis spinosa* is not very sensitive to pests and pathogens when growing in wilderness (Sozzi and Vicente, 2006). Caper diseases have never been considered as limiting factors for this crop, probably because of the low production density. However, Caper can be attacked by wide range of species including insects, viruses and fungi (Infantino et al., 2007; Table 2).

**Table 2:** Vulnerability of *Capparis spinosa* L. to pests and diseases.

**Category**	**Pathogen**	**Plant parts affected**	**Damages**	**Control**	**References**
Virus	Caper Latent Virus (CapLV)	Leaves	Asymptomatic	Understanding the epidemiology of each caper viruses Developing certification protocol for virus testing Obtaining small-scale production for virus-free seedlings	Ciferri, 1949; Di Franco and Gallitelli, 1985; Gallitelli and Di Franco, 1987; Adams et al., 2004; Tomassoli et al., 2005; Infantino et al., 2007
	Eggplant Mottled Dwarf Virus (EMDV)		Clearing, yellowing veins Necrosis Curling leaves Shortened internodes Severe dwarfing Decreasing yields.		
	Cucumber Mosaic Virus (CMV)		Mosaic leaves Chlorosis Mottled leaves Vein banding Yellow Spots		
	Co-infection CMV and EMDV or CapLV		Thickening, malformation of leaves Stunting of the plant		
Fungi	*Fusarium* spp.		Rotting of cuttings Damping-off of seedlings	Avoidance of excessive watering Use of steril soil	Lorente and Vicente, 1985
	*Sclerotium rolfsii*	Branches	Yellowing and wilting of branches Death of affected shoots	Removal of crop debris Weed control Reduction of stress factors	Infantino et al., 2006
	*Leveillula taurica*	Leaves, petioles, branches	Chlorosis Necrosis Defoliation Production of conidiophores	Sulphur-based fungicides Humidity reduction	Gupta and Bhardwaj, 1998; Kavak, 2004; Infantino et al., 2007
	*Albugo capparidis*	Mainly leaves and flowers	Whitre rust Hypertrophy of leaves, flowers, peduncles Floral abortion.	Destroying infected plants	Ciferri, 1949; Infantino et al., 2007
Insect Pests	^*^Acalles barbarus Lucas	Roots	Slender mines in the woods	–	Liotta, 1977
	^*^Phyllotreta latevittata Kutsch	Leaves	Circular gouges	–	Longo, 1996
	^**^:*Bagrada hilaris* (Burmeister) Nezara viridula (L.) Eurydema ventrale Kol. Eurydema ornata L. Holcostethus punctatus L. Carpocoris lunula F.	Leaves, buds, fruits	Yellowing spots and chlorosis Hollowing out plant parts Deformation	Insecticides (pyrethroids, organophosphates, cabamates) Some cultural practices like breaking uo the groundand destroying residues of alternative host plants	Colazza et al., 2004
	^***^: *Bemisia tabaci Aleurolobus niloticus Priesner and Hosny Brevicoryne brassicae (L.) Aspidiotus nerii Bouchè Planococcus citri Risso*	Leaves, stems	Yellowish spots and deformation Loss of vigor and leaves Death of plant	Spraying mineral oils	Rapisarda, 1985; Longo, 1996; Bayhan et al., 2006; Peri et al., 2006
	^****^: Pieris brassicae L. Pieris rapae (L.) Colotis evagore Lucas Anaphaeis aurota F. Colotis fausta fausta Olivier Lepidoptera Colotis liagore Klug. Cydia capparidana (Zel.) Lampides boeticus L.	Leaves, buds	Holes in leaves Deformation and abortion of buds	Insecticides based on *Bacillus thuringiensis*	Pittaway, 1979; Murzin, 1986; Fernández Garica, 1988; Jordano Barbudo et al., 1988; Kontaxis, 1990; Longo, 1996; Bayhan et al., 2006; Peri et al., 2006
	^*****^: Capparimyia savastani (Martelli) Asphondylia gennadii (Marchal) Capparimyia savastani(Martelli)	Buds, fruits	Deformation and abortion	“lur and kill” strategy with pyrethroids Cultural practices	Harris, 1975; Orphanides, 1975; Rangarajan and Mahadewan, 1975; Donati and Belcari, 2003; Bayhan et al., 2006; Peri et al., 2006

* *Cloeptera*,

** *Heteroptera*,

*** *Homoptera*,

**** *Lepidoptera*,

***** *Diptera, –, not available*.

### Economic value

The main economic importance of caper lays in dealing with flower buds, generally known in the market under the name of “capers” or “caper berry” which are the subject of considerable trade at an international level. Global caper production progressively increased at an annual growth rate of 6%. About 60 countries trade capers and the USA is considered as the most important consumer where the price reaches 25 US$/kg (ready for consumption). In the Balkans region, total production costs of caper represent less than 10% only of its selling price in the US markets. In Tunisia, the species is associated to a high socio-economic value especially for the rural farmers in the Northern country. The Chinese are earning an annual profit of 3 million US$ from this single specie (Saadaoui et al., 2011). More recently, *C. spinosa* is suggested to uplift the socio-economic level in the Kingdom of Saudi Arabia, in Lebanon, Syria and other Mediterranean countries (Sher and Alyemeni, 2010).

## Phytochemical composition and activities

### Extracts

*Capparis spinosa* has been investigated for its biochemical contents, which are affected by multiple factors such as geographical and environmental conditions, harvest date and size, preservation procedures, genotype, and processing methods of extraction (Sozzi and Vicente, 2006; Tlili et al., 2010). Capers are rich in phenolic compounds and flavonoids as reported in several studies (Table 3). Such secondary metabolites generally play a role in abiotic stress responses widely associated with tolerance to heat (Wahid, 2007). For instance, total phenolics ranged from 21.42 to 27.62 mg Gallic Acid Equivalent (GAE)/g of dry weight (DW) in caper leaves methanol extract taken from different sites in India. Caper leaves aqueous extract from Tunisia recorded total phenolics of 33.55 mg GAE/g DW and buds aqueous extracts contained 67.29 mg GAE/g DW, while 427.27 mg GAE/g DW of total phenolics was quantified in hydroethanolic extract of leaves. Iranian roots and fruits aqueous extracts contained 15.4 and 17.2 mg GAE/g DW respectively, lower than root ethyl acetate extracts containing 37.2 mg GAE/g DW and fruit ethanol extract containing 34.2 mg GAE/g DW.

**Table 3:** Chemical composition of the extracts from different organs of *Capparis spinosa* L.

**Organs**	**Extracts**	**Chemical constituents**	**Location**	**References**
Fruits	ME	(6S)-hydroxy-3-oxo-α-ionol glucoside,Corchoionoside C, prenyl glucoside, indol-3-acetonitrile glycoside, capparilloside A, capparilloside B.	Turkey	Calis et al., 2002
	AE	Flazin, guanosine, capparine A, capparine B, 1-H-Indole-3-carboxaldehyde, 4-hydroxy-1H-indole-3-carboxaldehyde, chrysoeriol, apigenin, kaempferol, thevetiaflavone, 5-hydroxymethylfuraldehyde, vanillic acid, cinnamic acid.	China	Haifeng et al., 2010
	EE/AF	Cappariside, 5-hydroxymethylfurfural, 5-hydroxymethyl furoic acid, 2-furoic acid.	China	Yang et al., 2010a
	EE/EF	Protocatechuic aldehyde, E-butenedioic acid, ethyl 3,4-dihydroxybenzoate, syringic acid, protocatechuic acid, vanillic acid, succinic acid, 4-hydroxybenzoic acid.		
	EE/AF	Capparisine A, capparisine B, capparisine C, 2-(5-hydroxymethyl-2-formylpyrrol-1-yl) propionic acid lactone, N-(30-maleimidy1)-5-hydroxymethyl-2-pyrrole formaldehyde.	China	Yang et al., 2010b
	EE	p-hydroxy benzoic acid, 5-(hydroxymethyl)furfural, bis(5-formylfurfural)ether, daucosterol, α-D-fructofuranosides methyl, uracil, stachydrine.	China	Feng et al., 2011
	EE/BF	Tetrahydroquinoline acid.	China	Zhang et al., 2014
	EE/EF	Racemic benzofuranone.		
	ME	Phenolics, flavonoids, carotenoids.	Bahrain	Allaith, 2014
Aerial Parts	ME	Quercetin 3-O-rutinoside, quercetin 3-O-glucoside, quercetin 3-O-glucoside-7-O-rhamnoside, Quercetin 3-O-[6^‴^ -α-L-rhamnosyl-6″ -β-D-glucosyl]-β-D-glucoside.	Egypt	Sharaf et al., 2000
	EE/HF	Terpene.	Jordan	Yang et al., 2010b; Muhaidat et al., 2013
	EE/AMF	Terpene, flavonoids.		
	EE/BF	Tanins, flavonoids, alkaloids.		
	EE/AF	Reducing sugar, flavonoids.		
Shoots and buds		Glucocapperin, glucoiberin, progoitrin, epiprogoitrin, sinigrin, gluconapoleiferin, glucoalyssin, gluconapin, 4-hydroxyglucobrassacin, glucobrassicanapin, glucobrassicin, gluconasturtiin.	Turkey	Matthäus and Ozcan, 2002
	EE/AMF	1-tetradecanol, methyl hexadecanoate, octadecanoic acid, 6,10,14-trimethyl-2-pentadecanone, β-sitosterol, glycerol monotetracostanoate, p-hydroxybenzaldehyde, ursolic acid, β-sitosterylglucoside, β-sitosterylglucoside-6′-octadecanoate.	Jordan	Khanfar et al., 2003
	EE/BF	4-coumaric acid, nicotinamide, cadabicine, isorhamnitine-3-O-rutinoside, rutin, stachydrine, 3-methyl-2-butenyl-β-glucoside.		
Leaves and Stems	EE	kaempferol 3-Rha-7-G, quercetin 3-Rut, quercetin 7-Rut, quercetin 3-G-7-Rhaw1.	China	Sharaf et al., 1997
Leaves and Flower buds	AE	5-Caffeoyl quinic acid, 1-Caffeoyl quinic acid, 5-p-Coumaroyl quinic acid, 4-Feruloyl quinic acid, Rutin, Quercetin 3-O-glc, Kaempferol 3-O-rutinoside, Methyl-quercetin-O-rutinoside, Kaempferol 3-O-glucoside, acids, flavonols.	Croatia	Kulisic-Bilusic et al., 2010
Roots	EE	Capparispine, Capparispine 26-O-b-D-glucoside, Cadabicine 26-O-b-D-glucoside hydrochloride.	China	Fu et al., 2008

*ME, Methanolic Extraction; AE, Aqueous Extraction; EE, Ethanolic Extraction; AF, Aqueous Fraction; EF, Ethyl acetateFraction; BF, Butanol Fraction; HF, Hexane Fraction; AMF, Aqueous Methanol Fraction*.

Total flavonoids registered 57 mg Quercetin Equivalent (QE)/g DW in hydroethanol extract of leaves and ranged from 2.6 to 6.96 mg QE /g DW in leaves methanol extract, whereas, 13.97 mg QE/ g DW and 25 mg QE/ g DW were found in leaves and flowers aqueous extracts respectively. Roots and fruits ethyl acetate extracts had a content of flavonoids of 95.5 and 18.1 mg QE/g respectively (Bhoyar et al., 2011; Mahboubi and Mahboubi, 2014; Akkari et al., 2016; Mansour et al., 2016). According to Inocencio et al. (2000), 10 g of commercial caper bud will provide 40 mg QE as aglycone in Mediterranean countries (Spain, Turkey, Morocco, Italy, Greece). *C. spinosa* is cited as a very good source of phenolic acids, alkaloids, flavonoids (rutin, quercetin, kaempferol) and glucosinolates (glucocapparin, glucoiberin, sinigrin, glucobrassicin) (Sozzi and Vicente, 2006; Kulisic-Bilusic et al., 2012; Francesca et al., 2016). The latter having hydrolysis products known as anti-cancer agents (Mithen et al., 2000).

The glucosinolate content of caper parts varies between 84 and 89%. Young shoots contain the highest amount of glucosinolate whereas the content in buds decreased as their size decreased. Glucocapperin (methyl glucosinolate) is the main glucosinolate of shoots and buds whereas indole glucosinolate (4-hydroxyglucobrassicin) is present in trace amounts in leaves and shoots (2.04 μmol/g), glucocapparin and glucocleomin appeared in seeds and leaves (Matthäus and Ozcan, 2002). Seeds are rich in oils, proteins, and fibers. Seed oils are adapted for feed and have a high content of linoleic, and oleic acids, sterols (namely, sitosterol, campesterol, stigmasterol and Δ^5^-avenasterol) and tocopherols (Akgül and Özcan, 1999; Matthäus and Özcan, 2005). In addition, the aliphatic (octadecanol as the major compound) and triterpenic (citrostadienol as the major compound) alcohol in the lipid unsaponifiable fraction were detected (Tlili et al., 2011b). These compounds can be integrated in cosmetic and pharmaceutical solutions. Seeds are rich in oils, proteins, and fibers. Seed oils are adapted for feed and food with a high content of linoleic, and oleic acids, sterols (namely, sitosterol, campesterol, stigmasterol and Δ^5^-avenasterol) and tocopherols (Akgül and Özcan, 1999; Matthäus and Özcan, 2005). In addition, the aliphatic (octadecanol as the major compound) and triterpenic (citrostadienol as the major compound) alcohol in the lipid unsaponifiable fraction were detected (Tlili et al., 2011b). These compounds can be integrated in cosmetic and pharmaceutical solutions.

The fruit constituents have been subject of interest in several studies in order to determine the biochemical content which is of great benefit in biology and food industries. From fruits of *C. spinosa*, 11 organic acid compounds and a new antioxidant active compound were isolated and identified and the structures of five novel alkaloids were determined (Yang et al., 2010a,b). Carotenoids and some terpenoids such as tocopherol stabilize and photo-protect the lipid–phase of the cell membrane providing great tolerance to increased temperatures (Velikova et al., 2005; Camejo et al., 2006). Aquaeous ethanolic fruit extracts contained flavonoids equivalent to rutin, phenolic compounds, tocopherol, carotenoid and vitamin C (Huseini et al., 2013). In addition to the known capparilloside A and stachydrine, an adenosine nucleoside, hypoxanthine and uracil were isolated from *C. spinosa* (Capparidaceae) fruits in China (Fu et al., 2007).

### Essential oils

The chemical composition of *C. spinosa* essential oils was subject to few studies (Table 4). Afsharypuor et al. (1998) determined 22 components in essential oil extracted from leaves, fruits and roots. The yield ranged from 0.02 to 0.9%. Fourteen components constituted leaf oil were detected (accounting for 91% of the total leaf oil composition); thymol (26.4%), isopropyl isothiocyanate (11%), 2-hexenal (10.2%), and butyl isothiocyanate (6.3%) represented the four major components. In the fruit oil only four components were detected (accounting for 98.5% of the total fruit oil composition); methyl isothiocyanate (41.6%) and isopropyl isothiocyanate (52.2%) were found as the two major components. Root components were represented mainly by methyl isothiocyanate (53.5%) and isopropyl isothiocyanate (31.4%). In Croatia, the essential oil of *C. spinosa* revealed that methyl isothiocyanate (92.06%) is the major compound in leaf and flower bud oils, in addition to benzyl isothiocyanate (0.74%), benzeneacetonitrile (0.40%), sec-butyl isothiocyanate (0.25%), and butyl isothioyanate (0.38%) (Kulisic-Bilusic et al., 2010). In the Eolian Archipelago, isothiocyanate is also a major component in caper, followed by benzyl isothiocyanate (Romeo et al., 2007). As to the Jordanian *C. spinosa*, essential oil is mostly represented by isopropyl isothiocyanate (28.92%), methyl isothiocyanate (25.6), and butyl isothiocyanate (16.65%) as major components (Muhaidat et al., 2013). Therefore, methyl isothiocyanate and isopropyl isothiocyanate are mainly present in fruits and roots, butyl-isothiocyanate is tissue-specific and is present in leaves but not in fruits and roots. Thiocyanate and isothiocyanate are break down products of glucosinolate as methyl glucosinolate (Glucocapperin) catalyzed by the enzyme myrosinase passes by the intermediate thiohydroximate and a rearrangement of the latter gives methyl isothiocyanate (Sozzi and Vicente, 2006).

**Table 4:** Chemical composition of the essential oil of *Capparis spinosa* L. obtained by hydrodistillation.

**References**	**Kulisic-Bilusic et al., 2010**	Afsharypuor et al., 1998			**Muhaidat et al., 2013**
**Collection location**	**Croatia**	Iran			**Jordan**
**Collection time**	**Jun-06**	Aug-95			**May-12**
**Species**	***C. spinosa* L**.	***C. spinosa*** L. var. ***mucronifolia*** (Boiss.)			***C. spinosa* L. var. *aravensis***
**Type of plant material**	**Floral buds and Leaves**	**Leaves**	**Fruits**	**Roots**	**Aerial parts**
**Yield**	**0.044% (w/w)**	**0.08% (v/w)**	**0.9% (v/w)**	**0.02% (v/w)**	**0.067% (w/w)**
**COMPONENT %**					
Methyl isothiocyanate	92.06		41.6	53.5	25.6
*Sec*-butyl isothiocyanate	0.25		2.2	0.6	
Butyl isothiocyanate	0.38	6.3			16.65
2-butenyl isothiocyanate					2.24
Benzene acetaldehyde	0.23				
Benzene acetonitrile	0.4				
*(E) β*-Ionone	0.5				
Methyl methylsalicilate	0.17				
Benzyl isothiocyanate	0.74				
3-Hexenyl benzoate	0.27				
3-Hexenyl benzoate	1.75				
Isopropyl isothiocyanate		11	52.2	31.4	28.92
2-Hexenal		10.2			
Unknown		4.4	2.5	10.1	
γ-Terpinene		4.7			
*n*-Dodecane		1.8			
Carvone		2.3			
Thymol		26.4			
*n*-Tetradecane		4.3			
Geranyl acetone		3.5			
*n*-Hexadecane		5.5			
Dill apiole		2.4			
Palmitic acid		4.7			
*n*-Eicosane		3.5			
3-p-menthene					3.08
3-methylthio-1-hexanal					2.03
Total %	96.75	91	98.5	95.6	

### Biological activities

Several researchers have reported different biological activities of *C. spinosa extracts* in various *in vivo* and *in vitro* test models. Certain pharmacological properties of great interest of *C. spinosa* had been identified and others are being studied (Moufid et al., 2015). It is worth noting that most of the evidences about biological activity and phytochemistry still derive from the analysis of wild plant material.

*C. spinosa* aqueous extracts displayed a significant anti-hyperglycemic activity and anti-obesity effects (Eddouks et al., 2004, 2005; Lemhadri et al., 2007). Indeed, consumption of caper fruit extracts by diabetic rats induced a decrease in both blood sugars and blood triglycerides (Rahmani et al., 2013). Likewise, a study on caper fruit ethanol extracts on type 2 diabetic patients in Iran showed significant decrease in fasting blood glucose levels and glycosylated hemoglobin and also a significant decrease in triglyceride level, thus assuring previous results on the anti-hyperglycemic and hypolipidemic effects of *C. spinosa* (Huseini et al., 2013). In multi-low dose streptozotocin-induced (MLDS) diabetic mice, a treatment with aqueous extract from fruits of *C. spinosa* promotes insulin sensitivity in peripheral tissues resulting in a lower endogenous glucose production (EGP) in treated than in untreated mice (Eddouks et al., 2017). Both leaf and root ethanolic extracts of *C. spinosa* showed inhibition of pancreatic α-amylase activities that could be involved in the control of blood sugar (Selfayan and Namjooyan, 2016).

Additionally Aghel et al. (2010) showed that the ethanolic root bark extract of *C. spinosa* has a significant dose-dependent protection against carbon tetrachloride and induced liver damage *in vivo*, in accordance with Gadgoli and Mishra (1999), who previously found that *p*-Methoxy benzoic acid isolated from the methanol soluble fraction of the aqueous extract of *C. spinosa* L. aerial part possesses an antihepatotoxic activity. Using Swiss albino mice intoxicated with trichloroacetic acid (TCA), a synergic effect between a mixture of C. spinosa leaves and honey to cope with the TCA hepatotoxicity has been shown (Alzergy et al., 2015). Cisplatin is one of the premium-choice drugs for the treatment of many cancers but it is not without drawbacks, principally toxicity to the liver and kidney. A recent work reported that methanolic extract of *C. spinosa* leaves significantly restored both the kidney and liver damages induced by cisplatin-treatment (Tlili et al., 2017).

Moreover, an *in vivo* study on murine indicated that the Tunisian *C. spinosa* leaf ethanol extract can stimulate melanogenesis in a dose-dependent manner without cytotoxicity. It can be useful in tanning and treating hair depigmentation (Matsuyama et al., 2009).

On the other hand, Panico et al. (2005) revealed the action exhibited by lyophilized extracts of *C. spinosa* (LECS) flower buds in developing novel anti-inflammatory/anti-degenerative agents that block the cartilage destruction during the inflammation *in vivo* and protect chondrocytes. More specifically, a recent study exhibited a better anti-inflammatory and analgesic effects for the fruits and stem-leaves of *C. spinosa* than those of the roots (Haifeng et al., 2010; Hong-Juan et al., 2014). The aqueous extracts from *C. spinosa* fruits were characterized as the best anti-inflammatory active fraction and also shown an anti-arthritic activity (Feng et al., 2011; Jiang et al., 2015).

Anti-inflammatory response of human peripheral blood mononuclear cells (PBMCs) induced by *C. spinosa* leaf extracts results from the control of cytokine gene expression (El Azhary et al., 2017). Cytokines constitute a category of small proteins (~5–20 kDa) that are important in cell signaling and inflammatory response. On PBMCs, *C. spinosa* extracts are able to suppress the expression of *IL-17*, coding for a pro-inflammatory cytokine, and promote the expression of *IL-4*, coding for an anti-inflammatory cytokine. Kulisic-Bilusic et al. (2010) isolated the essential oil of *C. spinosa* flower buds and leaves and proved its antioxidant activity by β-carotene bleaching method and thiobarbituric acid reactive species essay. Moreover, this same essential oil and the aqueous infusion of the same plant parts showed anti-proliferative activity on colon cancer cells by decreasing the activation of nuclear factor NF-Kappa B (aqueous infusion had more inhibition activity than essential oil) and arresting the cell cycle at G2/M phase (Kulisic-Bilusic et al., 2012).

Great anti-oxidant activity was demonstrated also in fresh caper berries methanolic extracts; more specifically, fruit on liver hepatocellular carcinoma cells (HepG2) (Yu et al., 2017). Pain associated with rheumatoid arthritis and osteoarthritis was soothed after single dose administration of caper root decoction and hydroalcoholic extracts to rat models (Maresca et al., 2016). The latter extract explored cardio protective effect by reducing the undesired apoptotic effect of an anti-cancer drug, doxorubicin (Mousavi et al., 2016).

Moreover, anti-fungal activity against *Valsa mali* and inhibition of HIV-1 reverse transcriptase activities were shown with a 38 kDa protein purified from *C. spinosa* fresh seeds from fruits. Inhibition of colon cancer MT29 cells, breast cancer MCF-7 cells and hepatoma HepG2 proliferation was also attributed to this caper protein (Lam and Ng, 2009; Lam et al., 2009).

Aqueous caper bud extracts alleviated neurodegeneration induced by lipopolysaccharide in rats thus showed protective effect against cognitive diseases, learning, and memory damage (Goel et al., 2016). The decoction of *C. spinosa* roots showed significant inhibition activity on the growth of *Deinococcus radiophilus* (Boga et al., 2011). The butanolic and aqueous methanolic extract fractions from Jordanian *C. spinosa* showed antibacterial activity against *Staphylococcus epidermis* (ATCC 12228), whereas petroleum ether, hexane and water fractions exhibited antibacterial activity against *Streptococcus faecalis* (ATCC 29212) (Muhaidat et al., 2013).

## Traditional uses

### Food and culinary

Caper is a potential source of valuable nutrients, since 100 g of caper fruit contain 67 mg calcium, 65 mg phosphorus, 9 mg iron, and 24.5 g protein.

Commercial capers are immature flower buds that can be pickled in salt or vinegar and used as an appetizer or condiment (Saadaoui et al., 2011). Hence, capers are included in hundreds of recipes due to their sharp piquant flavor owed to a complex organoleptic profile (Brevard et al., 1992) and are used as a seasoning to add pungency to sauces (e.g., tartare, remoulade, ravigote etc.,) dressings and salads (e.g., caponata, a cold eggplant salad with olives and capers), cold dishes and sauces served with salmon, herring, pasta and pizzas, cheeses, lamb, mutton, pork and chicken preparations (Sozzi and Vicente, 2006).

Unripe fruits called caper berries are also pickled and used as spices and condiments (Rivera et al., 2003). Food industries also use extracts from Caper buds and ripened fruits as flavor agents (Aliyazicioglu et al., 2015).

### Medicinal

*Capparis spinosa* L. (Capparidaceae) is one of the medicinal plants that have been widely used in the traditional medicine during successive civilizations to cure various health disorders and illnesses. A wide range of therapeutic benefits are credited to caper extracts such as anti-hypertensive (Ali et al., 2007), anti-hepatotoxic (El Tanbouly et al., 1989; Gadgoli and Mishra, 1999), anti-diabetic (Kazemian et al., 2015; Mollica et al., 2017; Vahid et al., 2017), anti-obesity (Lemhadri et al., 2007), bronchorelaxant (Benzidane et al., 2013), anti-allergic and anti-histaminic (Angelini et al., 1991; Trombetta et al., 2005), anti-inflammatory (Al-Said et al., 1988; Zhou et al., 2010) or anti-biotic (Abraham et al., 2011; Mahboubi and Mahboubi, 2014) properties.

Iranian people used the root, fruit and plant bark of *C. spinosa* as diuretics and tonics against malaria and joint disease (Hooper, 1937; Afsharypuor et al., 1998). In Pakistan, leaves of *C. spinosa* are used as analgesic, anti-hemorrhoid, anti-rheumatic, aperients, deobstruent, depurative and diuretic (Chopra et al., 1986). In India, buds and roots of *C. spinosa* are useful in the treatment of boils, while leaves are used as counter-irritant and as a cataplasm in swellings. Roots are used to treat fever, rheumatism, paralysis, toothache and kill worms in the ear. Bark is used against coughs, asthma and inflammation (Wealth of India, 1992). The stem-leaves, fruits, and roots have been used for the treatment of rheumatoid arthritis and gout in traditional medicine in China (Feng et al., 2011).

The root bark of Caper has been used as an analgesic and carminative agent and possesses antihypertensive activity as well (Eddouks et al., 2004; Lemhadri et al., 2007). Decoctions from root bark are also used to treat dropsy, anemia and rheumatism. Herbal tea made from root and young shoots is of benefit for the treatment of rheumatism, stomach and intestinal disorders. In the folklore of the central region of Saudi Arabia, along with *C. spinosa* diuretic and body tonic utilization, pastes prepared from the root bark are used externally to treat swollen joints, skin rashes and dry skin. During the last decade, some cosmetic products derived from *C. spinosa* fruit extract (e.g., Gatuline® Derma-Sensitive; SKIN MOON®; SKIN SAVE®) were commercialized, claiming skin protection and anti-aging or anti-inflammatory properties. Herbal tea prepared by *C. spinosa* buds and leaves is found to be a popular remedy against cold and related infections, also decoction of buds and leaves is used internally for curing gastrointestinal infections, diarrhea, and dysentery and also useful for the removal of kidney stones (Sher and Alyemeni, 2010). In Morocco, unopened buds are used externally to treat eye infections and prevent cataracts while caper dried fruits are meant to cure hypertension and diabetic complications when taken orally with a glass of water (Jouad et al., 2001; Eddouks et al., 2002).

Recent review articles provide a detailed overview of the state of the art in the field of medicinal/pharmaceutical properties of *C. spinosa* (Moufid et al., 2015; Anwar et al., 2016; Nabavi et al., 2016; Rahnavard and Razavi, 2016).

## Conclusion

This review encourages further studies on *C. spinosa* in the East Mediterranean countries to face the changing environment, climate-mediated transition of agriculture and to promote its nutritional and health benefits. This plant has various medicinal, culinary, agronomic and economic values. Caper cultivation could be a good solution for implementing needed novel agricultural practices for climate risk management and production sustainability. Its remarkable ability to adapt to different climates call upon to integrate *C. spinosa* in the long-term agricultural strategy to cope with larger impacts of climate changes in the future.

The establishment of genetic data for taxonomic identification and productivity is a priority research need for caper. Genetic variability for tolerance to heat stress should be exploited in order to screen germplasm and select cultivars with high temperature tolerant genotypes. High genetic potential can also be exhibited by selection of hybrids and induced crossings.

The identification of molecular markers correlated with phenotypic traits of caper will be a future tool to promote stress-related breeding programs, as well as an integrative view of the biology of the species and its evolution.

The traditional medicinal knowledge and the biological studies have to find ways to enlarge the benefits and the capacities of this natural plant resource.

Finally, this plant could be integral part of family farming and value chain products in the Mediterranean contributing enormously to socio-economic development.

## Author contributions

SC, wrote the paper. AA, ME, LC, NO, and LR made some corrections and additional contributions.

### Conflict of interest statement

The authors declare that the research was conducted in the absence of any commercial or financial relationships that could be construed as a potential conflict of interest.

## References

[B1] Abraham S. V. P. I. Palani A. Ramaswamy B. R. Shunmugiah K. P. Arumugam V. R. (2011). Antiquorum sensing and antibiofilm potential of *Capparis spinosa*. Arch. Med. Res. 42, 658–668. 10.1016/j.arcmed.2011.12.002 22222491

[B2] Adams M. J. Antoniw J. F. Bar-Joseph M. Brunt A. A. Candresse T. Foster G. D.. (2004). The new plant virus family *Flexiviridae* and assessment of molecular criteria for species demarcation. Arch. Virol. 149, 1045–1060. 10.1007/s00705-004-0384-x 15098118

[B3] Afsharypuor S. Jeiran K. Jazy A. A. (1998). First investigation of the flavor profiles of leaf, ripe fruit and root of *Capparis spinosa* var. mucronifolia from Iran. Pharm. Acta. Helv. 5, 307–309. 10.1016/S0031-6865(97)00023-X

[B4] Aghel N. Rashidi I. Mombeini A. (2010). Hepatoprotective activity of *Capparis spinosa* root bark against CCl4 induced hepatic damage in mice. Iran. J. Pharm. Res. 6, 285–290.

[B5] Ahmed M. (1986). Vegetation of some foothills of Himalayan range in Pakistan. Pak. J. Bot. 18, 261–269.

[B6] Akgül A. Özcan M. (1999). Some compositional characteristics of Capers (*Capparis* spp.) seed and oil. Grasas Aceites. 50, 49–52. 10.3989/gya.1999.v50.i1.635

[B7] Ali Z. N. Eddouks M. Michael J. B. (2007). Cardiovascular effect of *Capparis spinosa* aqueous extract. Part III: antihypertensive effect in spontaneously hypertensive rats. Am. J. Pharmacol. Toxicol. 2, 111–115. 10.3844/ajptsp.2007.111.115

[B8] Akkari H. B'chir F. Hajaji S. Rekiki M. Sebai E. Hamza H. (2016). Potential anthelmintic effect of *Capparis spinosa* (Capparidaceae) as related to its polyphenolic content and antioxidant activity. Vet. Med. 61, 308–316. 10.17221/169/2015-VETMED

[B9] Aliyazicioglu R. Tosun G. Eyupoglu E. (2015). Characterisation of volatile compounds by spme and gc-fid/ms of capers (*Capparis spinosa* L.). Afr. J. Agric. Res. 10, 2213–2217.

[B10] Alkire B. (1998). Capers. Center for New Crops and Plants Products. West Lafayette, IN: Purdue University.

[B11] Allaith A. A. A. (2014). Assessment of the antioxidant properties of the caper fruit (*Capparis spinosa* L.) from Bahrain. J. Assoc of Arab Univ. Basic Appl. Sci. 19, 1–7. 10.1016/j.jaubas.2014.07.001

[B12] Al-Safadi B. Elias R. (2011). Improvement of caper (*Capparis spinosa* L.) propagation using *in vitro* culture and gamma irradiation. Sci. Hortic. 127, 290–297. 10.1016/j.scienta.2010.10.014

[B13] Al-Safadi B. Faouri H. Elias R. (2014). Genetic diversity of some *Capparis* L. species growing in Syria. Braz. Arch. Biol. Technol. 57, 916–926. 10.1590/S1516-8913201402549

[B14] Al-Said M. S. Abdelsattar E. A. Khalifa S. I. El-Feraly F. S. (1988). Isolation and identification of an anti-inflammatory principle from *Capparis spinosa*. Die Pharmazie 43, 640–641. 3244735

[B15] Al-Yemeni M. N. Zayed K. M. (1999). Ecology of some plant communities along Riyadh Al-Thumamah Road, Saudi Arabia. Saudi. J. Biol. Sci. 6, 9–26.

[B16] Alzergy A. A. Elgharbawy S. M. Mahmoud G. S. Mahmoud M. R. (2015). Role of *Capparis spinosa* in ameliorating trichloroacetic acid induced toxicity in liver of Swiss albino mice. Life Sci. J. 12, 26–39.

[B17] Andrade G. Esteban E. Velasco L. Lorite M. J. Bedmar E. J. (1997). Isolation and identification of N2-fixing microorganisms from the rhizosphere of *Capparis spinosa* (L.). Plant Soil. 197, 19–23. 10.1023/A:1004211909641

[B18] Angelini G. Vena G. A. Filotico R. Foti C. Grandolfo M. (1991). Allergic contact dermatitis from *Capparis spinosa* L. applied as wet compresses. Contact Derm. 24, 382–383. 10.1111/j.1600-0536.1991.tb01764.x 1893693

[B19] Anwar F. Muhammad G. Hussain M. A. Zengin G. Alkharfy K. M. Ashraf M. (2016). *Capparis spinosa* L.: a plant with high potential for development of functional foods and nutraceuticals/pharmaceuticals. Int. J. Pharm. 12, 201–219. 10.3923/ijp.2016.201.219

[B20] Bahrani M. J. Ramazani G. M. Shekafandeh A. Taghvaei M. (2008). Seed germination of wild Caper (*Capparis spinosa* L., var. parviflora) as affected by dormancy breaking treatments and salinity levels. Seed Sci. Technol. 36, 776–780. 10.15258/sst.2008.36.3.27

[B21] Barbera G. (1991). Le câprier (*Capparis* spp.), in Programme de Recherche Agrimed, ed Guiseppe B. (Luxembourg: Commission des Communautés européennes L- 2920), 62.

[B22] Barbera G. Di Lorenzo R. (1984). The caper culture in Italy. Acta Hortic. 144, 167–171. 10.17660/ActaHortic.1984.144.21

[B23] Bayhan E. Ulusoy M. R. Brown J. K. (2006). Host range, distribution, and natural enemies of Bemisia tabaci ‘B biotype’ (Hemiptera: Aleyrodidae) in Turkey. J. Pest. Sci. 79, 233–240. 10.1007/s10340-006-0139-4

[B24] Benzidane N. Charef N. Krache I. Baghiani A. Arrar L. (2013). *In vitro* bronchorelaxant effects of *Capparis Spinosa* aqueous extracts on rat trachea. J. Appl. Pharm. Sci. 3, 85–88

[B25] Bhoyar M. S. Mishra P. G. Naik K. P. Murkute A. A. Srivastavar B. R. (2012). Genetic variability studies among natural populations of *Capparis spinosa* from cold arid desert of trans-himalayas using DNA markers. Natl. Acad. Sci. Lett. 35, 505–515. 10.1007/s40009-012-0086-y

[B26] Bhoyar M. S. Mishra P. G. Naik K. P. Srivastava R. (2011). Estimation of antioxidantactivity and total phenolics among natural populations of Caper (*Capparis spinosa*) leaves collected from cold arid desert of trans-Himalayas. Aust. J. Crop. Sci. 5, 912–919.

[B27] Bita C. E. Gerats T. (2013). Plant tolerance to high temperature in a changing environment: scientific fundamentals and production of heat stress-tolerant crops. Front. Plant Sci. 4:273. 10.3389/fpls.2013.00273 23914193 PMC3728475

[B28] Boga C. Forlani L. Calienni R. Hindley T. Hochkoeppler A. Tozzi S.. (2011). On the antibacterial activity of roots of *Capparis spinosa* L. Nat. Prod. Res. 25, 417–421. 10.1080/14786419.2010.487189 21328135

[B29] Bond R. E. (1990). The caper bush. Herbarist 56, 77–85.

[B30] Bounous G. Barone E. (1989). Il cappero: prospettive di sviluppo di specie legnose per le zone aride e semi-aride del meridione e nuovi criteri di utilizzo. Terra e Sole. 44, 733–735.

[B31] Brevard H. Brambilla M. Chaintreau A. Marion J. P. Diserens H. (1992). Occurrence of elemental sulphur in capers (*Capparis spinosa* L.) and first investigation of the flavour profile. Flav. Frag. J. 7, 313–321. 10.1002/ffj.2730070605

[B32] Caglar C. Caglar S. Ergin O. Yarim M. (2005). The influence of growth regulators on shoot proliferation and rooting of *in vitro* propagated caper. J. Environ. Biol. 26, 479–485. 16334286

[B33] Caliş I. Kuruüzüm A. Lorenzetto P. A. Rüedi P. (2002). (6S)-Hydroxy-3-oxo-a-ionol glucosides from *Capparis spinosa* fruits. Phytochemistry 59, 451–457. 10.1016/S0031-9422(01)00399-5 11830166

[B34] Camejo D. Jiménez A. Alarcón J. J. Torres W. Gómez J. M. Sevilla F. (2006). Changes in photosynthetic parameters and antioxidant activitis following heat-shock treatment in tomato plants. Funct. Plant Biol. 33, 177–187. 10.1071/FP05067 32689224

[B35] Carra A. Del Signore M. B. Sottile F. Ricci A. Carimi F. (2011). Potential use of new diphenylurea derivatives in micropropagation of *Capparis spinosa* L. Plant Growth Regul. 66, 229–237. 10.1007/s10725-011-9645-3

[B36] Carra A. Sajeva M. Abbate L. Siragusa M. Sottile F. Carimi F. (2012). *In vitro* plant regeneration of caper (*Capparis spinosa* L.) from floral explants and genetic stability of regenerants. Plant Cell Tissue Organ Cult. 109, 373–381. 10.1007/s11240-011-0102-9

[B37] Chalak L. Elbitar A. (2006). Micropropagation of *Capparis spinosa* L. subsp. *rupestris* sibth & Sm. by nodal cuttings. Indian J. Biotechnol.. 5, 555–558.

[B38] Chalak L. Elbitar A. Cordahi N. Hage C. Chehade A. (2003). *In vitro* propagation of *Capparis spinosa* L. Acta Hortic. 616, 335–338. 10.17660/ActaHortic.2003.616.48

[B39] Chalak L. Perin A. Elbitar A. Chehade A. (2007). Phenotypic diversity and morphological characerization of *Capparis spinosa* L. in Lebanon. Biol. Tunisie 4, 28–32.

[B40] Chopra R. N. Nayar S. Chopra I. C. (1986). Glossary of Indian Medicinal Plants (with Supplement). New Delhi: Council of Scientific and Industrial Research.

[B41] Ciferri R. (1949). Rassegna di parassiti e malattie del cappero (*Capparis spinosa* L.) in Italia. Notiziario sulle Malattie delle Piante 3, 33–35.

[B42] Colazza S. Guarino S. Peri E. (2004). *Bagrada hilaris* (Burmeister) (Heteroptera: Pentatomidae) fitofago dannoso al cappero nell'isola di Pantelleria. Inf. Fitopatol. 53, 30–34.

[B43] Danin A. (2010). *Capparis* in the East Mediterranean countries. Fl. Medit. 20, 179–185.

[B44] Di Franco A. Gallitelli D. (1985). Rhabdovirus-like particles in caper leaves with vein yellowing. Phytopathol. Mediterr. 24, 234–236.

[B45] Donati M. Belcari A. (2003). A note on insect pests of the caper plant in Jordan, with special reference to *Capparimyia savastani* (Martelli) (Diptera, Tephriti-dae). Stud. dipterol. 10, 395–400.

[B46] Eddouks M. Lemhadri A. Hebi M. Hidani A. E. Zeggwagh N. A. El Bouhali B.. (2017). *Capparis spinosa* L. aqueous extract evokes antidiabetic effect in streptozotocin-induced diabetic mice. Avicenna J. Phytomed. 7, 191–198. 28348974 PMC5355824

[B47] Eddouks M. Lemhardi A. Michel J. B. (2004). Caraway and caper potential antihyperglycaemic plants in diabetic rats. J. Ethnopharmacol. 94, 143–148. 10.1016/j.jep.2004.05.006 15261975

[B48] Eddouks M. Lemhardi A. Michel J. B. (2005). Hypolipidemic activity of aqueous extract of *Capparis spinosa* L. in normal and diabetic rats. J. Ethnopharmacol. 98, 345–350. 10.1016/j.jep.2005.01.053 15814271

[B49] Eddouks M. Maghrani M. Lemhadri A. Ouahidi M. L. Jouad H. (2002). Ethnopharmacological survey of medicinal plants used for the treatment of diabetes mellitus, hypertension and cardiac diseases in the south-east region of Morocco (Tafilalet). J. Ethnopharmacol. 103, 82–97. 10.1016/S0378-8741(02)00164-2 12241983

[B50] El Azhary K. Jouti N. T. El Khachibi M. Moutia M. Tabyaoui I. El Hou A.. (2017). Anti-inflammatory potential of *Capparis spinosa* L. in vivo in mice through inhibition of cell infiltration and cytokine gene expression. BMC Complement Altern. Med. 17:81 10.1186/s12906-017-1569-7 28143472 PMC5282892

[B51] El Tanbouly N. Joyeux M. Hanna S. Fleurentin J. El Alfy T. Anton R. (1989). Antihepatotoxic effect of aqueous extracts from *Capparis spinosa*. Planta Medica. 55, 95–95. 10.1055/s-2006-961847

[B52] Faran M. (2014). Capparis spinosa - the plant on the wall, in Medicinal and Aromatic Plants of the Middle-East (Medicinal and Aromatic Plants of the World), eds Yaniv Z. Dudai N. (Dordrecht: Springer Netherlands), 59–65.

[B53] Feng X. Lu J. Xin H. Zhang L. Wang Y. Tang K. (2011). Anti-arithritic active fraction of *Capparis spinosa* L. fruits and its chemcial constituents. Yakugaki Zasshi 13, 423–429. 10.1248/yakushi.131.423 21372539

[B54] Fernández Garica E. (1988). Spring and summer hosts for *Pieris rapae* in Southern Spain with special attention to *Capparis spinosa*. Entomol. Exp. Appl. 48, 173–178. 10.1111/j.1570-7458.1988.tb01161.x

[B55] Fici S. (2001). Intraspecific variation and evolutionary trends in *Capparis spinosa* L. (Capparaceae). Plant Syst. Evol. 228, 123–141. 10.1007/s006060170024

[B56] Fici S. (2014). A taxonomic revision of the *Capparis spinosa* group (Capparaceae) from the Mediterranean to Central Asia. Phytotaxa. 174, 1–24. 10.11646/phytotaxa.174.1.1

[B57] Fici S. (2015). A taxonomic revision of the *Capparis spinosa* group (Capparaceae) from eastern Africa to Oceania. Phytotaxa. 203, 24–36. 10.11646/phytotaxa.203.1.2

[B58] Francesca N. Barbera M. Martorana A. Saiano F. Gaglio R. Aponte R.. (2016). Optimised method for the analysis of phenolic compounds from caper (*Capparis spinosa* L.) berries and monitoring of their changes during fermentation. Food Chem. 196, 1172–1179 10.1016/j.foodchem.2015.10.045 26593604

[B59] Fu X. P. Aisa A. H. Abdurahim M. Yili A. Aripova F. S. Tashkhodzhaev B. (2007). Chemical composition of *Capparis spinosa* fruit. Chem. Nat. Compd. 43, 181–183. 10.1007/s10600-007-0074-5

[B60] Fu X. P. Wu T. Abdurahim M. Su Z. Hou L. X. Aisia A. H. (2008). New spermidine alkaloids from *Capparis spinosa* roots. Phytochem. Lett. 1, 59–62. 10.1016/j.phytol.2008.01.001

[B61] Gadgoli C. Mishra S. H. (1999). Antihepatotoxic activity of *p*-methoxy benzoic acid from *Capparis spinosa*. J. Ethnopharmacol. 66, 187–192. 10.1016/S0378-8741(98)00229-3 10433476

[B62] Gallitelli D. Di Franco A. (1987). Characterization of caper latent virus. J. Fitopatol. 119, 97–105 10.1111/j.1439-0434.1987.tb00471.x

[B63] Gan L. Zhang C. Yin Y. Lin Z. Huang Y. Xiang J. (2013). Anatomical adaptations of the xerophilous medicinal plant, *Capparis spinosa*, to drought conditions. Hortic. Environ. Biotechnol. 54, 156–161. 10.1007/s13580-013-0162-3

[B64] Goel A. Digvijaya Garg A. Kumar A. (2016). Effect of *Capparis spinosa* Linn. extract on lipopolysaccharide-induced conginitive impairments in rats. Indian J. Exp. Biol. 54, 126–132. 26934780

[B65] González S. (1973). La alcaparra: características y comercialización. Agricultura 495, 422–425.

[B66] Gorini F. (1981). Schede orticole. 6. Ortaggi da infiorescenze. 6.4. Cappero. Informatore di Ortoflorofrutticoltura. 6, 3–4.

[B67] Güleryüz M. Özkan G. Ercisli S. (2009). Caper (*Capparis* spp.) growing techniques and economical importance, in 1st International Symposium on Sustainable Development (Sarajevo), 94–97.

[B68] Gull T. Anwar F. Sultana B. Alcayde C. A. M. Nouman W. (2015). *Capparis* species: a potenial source of bioactives and high-value comonents: a review. Ind. Crops Prod. 67, 81–96. 10.1016/j.indcrop.2014.12.059

[B69] Gupta A. K. Bhardwaj L. N. (1998). Additional host of *Leveillula taurica (Lev.)* G. Arnaud from India. Indian Phytopathol. 51, 104–106.

[B70] Haifeng Z. Renji J. Jie K. Xiaoling H. Yan L. Changlong Z. (2010). Antiinflammatory effects of caper *(Capparis spinosa L.)* fruit aqueous extract and the isolation of main phytochemicals. J. Agric. Food Chem. 58, 12717–12721. 10.1021/jf1034114 21105652

[B71] Hall C. J. Systma J. K. Iltis H. H. (2002). Phylogeny of Capparaceae and Brassicaceae *based* on chloroplast seauence data. Am. J. Bot. 89, 1826–1842. 10.3732/ajb.89.11.1826 21665611

[B72] Hansen J. (1991). The Palaeoethnobotany of Franchthi Cave. Bloomington: Indiana University Press.

[B73] Harris K. M. (1975). The taxonomic status of the carob gall midge, *Asphondylia gennadii* (Marchal), comb. *n*. (Diptera, Cecidomyiidae), and of other Asphondylia species recorded from Cyprus. Bul. Entomol. Res. 65, 377–380. 10.1017/S0007485300006040

[B74] Hatfield J. L. Prueger J. H. (2015). Temperature extremes: effect on plant growth and development. Weather Clim. Extremes 10, 4–10. 10.1016/j.wace.2015.08.001

[B75] Heywood V. H. (1964). *Capparis* L., in Flora Europaea, ed Heywood V. H. Tutin T. G. Bugres N. A. Moore D. M. Valentine D. H. Waletrs S. M. (Cambridge Cambridge University Press), 259.

[B76] Higton R. N. Akeroyd J. R. (1991). Variation in *Capparis spinosa* L. in Europe. Bot. J. Linn. Soc. 106, 104–112.

[B77] Hillman G. C. (1989). Late Palaeolithic plant foods from Wadi Kubbaniya in Upper Egypt: dietary diversity, infant weaning, and seasonality in a riverine environment, in Foraging and Farming: The Evolution of Plant Exploitation, ed Harris D. R. Hillman G. C. (London: Unwin Hyman), 207–239.

[B78] Hong-Juan L. Tao Y. Xue-Mei C. Chang-Hong W. (2014). Comparative evaluation of anti-inflammatory and analgesic activities of various medicinal parts of *Capparis spinosa*: a consideration of ecological environment and resource conservation. Indian J. Med. Res. Pharm. Sci. 4, 53–59.

[B79] Hooper D. (1937). Useful Plants and Drugs of Iran and Iraq. Chicago: Field Museum of Natural History.

[B80] Howden M. S. Soussana J. F. Tubiello N. F. Chhetri N. Dunlop M. Holger Meinke H. (2007). Adapting agriculture to climate change. Proc. Natl. Acad. Sci. U.S.A. 104, 19691–19696. 10.1073/pnas.0701890104 18077402 PMC2148359

[B81] Huseini F. H. Hasani-Rnjbar S. Nayebi N. Heshmat R. Sigaroodi K. F. Ahvazi M.. (2013). *Capparis spinosa* L. (Caper) fruit extract in treatment of type 2 diabetic patients: a randomized double-blind placebo-controlled clinical trial. Complement Ther. Med. 21, 447–452. 10.1016/j.ctim.2013.07.003 24050578

[B82] Infantino A. Pucci N. Di Giambattista G. Tomassoli L. (2006). *Capparis spinosa*- a new host for Sclerotium rolfsii. Plant Pathol. 55, 580 10.1111/j.1365-3059.2006.01376.x

[B83] Infantino A. Tomassoli L. Peri E. Colazza S. (2007). Viruses, fungi and insect pests affecting caper. Eur. J. Plant Sci. Biotech. 1, 170–179.

[B84] Inocencio C. Alcaraz F. Calderon F. Obon C. Rivera D. (2002). The use of floral characters in *Capparis* sect. Capparis' to determine the botanical and geographical origin of capers. Eur. Food Res. Technol. 214, 335–339. 10.1007/s00217-001-0465-y

[B85] Inocencio C. Alcaraz F. Tomas-Barberan F. (2000). Flavonoid content of commercial Capers (*Capparis spinosa, C. sicula* and *C. orientalis*) produced in Mediterranean countries. Eur. Food Res. Technol. 212, 70–74. 10.1007/s002170000220

[B86] Inocencio C. Cowan S. R. Alcaraz F. Rivera D. Fay F. M. (2005). AFLP fingerprinting in *Capparis* subgenus *Capparis* related to the commercial sources of capers. Genet. Resour. Crop. Evol. 52, 137–144. 10.1007/s10722-003-4432-2

[B87] Inocencio C. Rivera D. Obon C. Alcaraz F. Barrena J. A. (2006). A systematic revision of *Capparis* section *Capparis* (Capparaceae). Ann. Missouri. Bot. Gard. 93, 122–149. 10.3417/0026-6493(2006)93[122:ASROCS]2.0.CO;2

[B88] Jacobs M. (1960). Capparidaceae. Flora Malesiana. 1, 61–105.

[B89] Jacobs M. (1965). The genus *Capparis* (Capparaceae) from the Indus to the Pacific. Blumea 12, 385–541.

[B90] Jiang H. E. Li X. Ferguson K. D. Wang Y. F. Liu C. J. Li C. S. (2007). The discovery of *Capparis spinosa* L. (Capparidaceae) in the Yanghai Tombs (2800 years b.p.), NW China, and its medicinal implications. J. Ethnopharmacol. 113, 409–420. 10.1016/j.jep.2007.06.020 17693045

[B91] Jiang S. S. Ma W. N. Lu W. J. Ma G. Z. (2015). Preliminary screening of anti- inflammatory active fractions from fruits of *Capparis spinosa* of uighur medicine. Chin. J. Expe. Trad. Med. Form. 4:041.

[B92] Jordano Barbudo D. Rodriguez Gonzalez J. Fernandez Haeger J. (1988). *Capparis spinosa* (Capparidaceae): on oviposition substrate for *Lampides boeticus* Linnaeus, in southern Spain (Lepidoptera: Lycaenidae). Nota Lepid. 10, 218–223.

[B93] Jouad H. Rhiouani H. El Hilaly J. M. Eddouks M. (2001). Ethnobotanical survey of medicinal plants used for the treatment of diabetes, cardiac and renal diseases in the North centre region of Morocco (Fez–Boulemane). J. Ethnopharmacol. 77, 175–182. 10.1016/S0378-8741(01)00289-6 11535361

[B94] Kala P. C. Mathur B. V. (2002). Patterns of plant species distribution in the Trans-Himalayan region of Ladakh, India. J. Veget. Sci. 13, 751–754. 10.1111/j.1654-1103.2002.tb02104.x

[B95] Kavak H. (2004). Epidemic outbreaks of powdery mildew caused by *Leveillula taurica* on *Capparis spinosa* in Turkey. Plant Pathol. 53:809 10.1111/j.1365-3059.2004.01072.x

[B96] Kazemian M. Abad M. Haeri M. R. Ebrahimi M. Heidari R. (2015). Anti-diabetic effect of *Capparis spinosa* L. root extract in diabetic rats. Avicenna J. Phytomed. 5:325. 26445712 PMC4587611

[B97] Khanfar M. A. Sabri S. S. Zarga M. H. Zeller K. P. (2003). The chemical constituents of *Capparis spinosa* of jordanian origin. Nat. Prod. Res. 17, 9–14. 10.1080/10575630290034302 12674136

[B98] Khouildi S. Pagnotta M. A. Tanzarella O. A. Ghorbel A. Porceddu E. (2000). Suitability of RAPD (random amplified polymorphic DNA) technique for estimating the genetic variation in natural genotypes of Tunisian and Italian caper (*Capparis spinosa* L.). Agricoltura-Mediterranea 130, 72–77.

[B99] Kontaxis D. G. (1990). Pest of caper, *Capparis spinosa*. Some new records for California. Phytopathology. 80:1026.

[B100] Kulisic-Bilusic T. Balzevic I. Dejanovic B. Milos M. Pifat G. (2010). Evaluation of the antioxidant activity of essential oils from caper (*Capparis spinosa*) and sea fennel (*Crithmum maritimum*) by different methods. J. Food Biochem. 34, 286–302. 10.1111/j.1745-4514.2009.00330.x

[B101] Kulisic-Bilusic T. Schmoller I. Schnäbele K. Siracusa L. Ruberto G. (2012). The anticarcinogenic potential of essential oil and aqueous infusion from caper (Capparis spinosa L.). Food Chem. 132, 261–267. 10.1016/j.foodchem.2011.10.074 26434289

[B102] Lam S. K. Han Q. F. Ng T. B. (2009). Isolation and characterization of a lectin with potentially exploitable activities from caper (*Capparis spinosa*) seeds. Biosci. Rep. 29, 293–299. 10.1042/BSR20080110 18847434

[B103] Lam S. K. Ng T. B. (2009). A protein with antiproliferative, antifungal and HIV-1 reverse transcriptase inhibitory activities from caper (*Capparis spinosa*) seeds. J. Phytomed. 16, 444–450. 10.1016/j.phymed.2008.09.006 19019643

[B104] Legua P. Martínez J. J. Melgarejo P. Martínez R. Hernández F. (2013). Phenological growth stages of caper plant (*Capparis spinosa* L.) according to the Biologische Bundesanstalt, Bundessortenamt and CHemical scale. Ann. Appl. Biol. 163, 135–141. 10.1111/aab.12041

[B105] Lemhadri A. Eddouks M. Sulpice T. Burcelin R. (2007). Antihyperglycaemic and anti-obesity effects of *Capparis spinosa* and *Chamaemelum nobile* aqueous extracts in HFD Mice. Am. J. Pharm. Toxicol. 2, 106–110. 10.3844/ajptsp.2007.106.110

[B106] Levizou E. Drilias P. Kyparissis A. (2004). Exceptional photosynthetic performance of *Capparis spinosa* L. under adverse conditions of Mediterranean summer. Photosynthetica 42, 229–235. 10.1023/B:PHOT.0000040594.85407.f4

[B107] Li Q. Yu L. Deng Y. Li W. Li M. Cao J. (2007). Leaf epidermal characters of *Lonicera japonica* and *Lonicera confusa* and their ecology adaptation. J. For. Res. 18, 103–108. 10.1007/s11676-007-0020-1

[B108] Liotta G. (1977). Acalles barbarus Lucas (s.l.) su Capparis spinosa L. a Pantelleria (Col. Curculionidae), Nota bio-etologica' (Summary in English). Nat. Sicil 1, 39–45.

[B109] Liu C. Xue P. G. Cheng B. Wang X. He J. Liu H. G.. (2015). Genetic diversity analysis of *Capparis spinosa* L. populations by using ISSR markers. Genet. Mol. Res. 14, 16476–16483. 10.4238/2015.December.9.19 26662446

[B110] Longo S. (1996). La mosca del cappero. Inf. Agrar. 52, 65–69.

[B111] Lorente F. L. Vicente M. P. (1985). La Tapenera o Alcaparra: Cultivo y Aprovechamiento. Madrid: Ministerio de Agricultura, Pesca y Alimentación.

[B112] Mahboubi M. Mahboubi A. (2014). Antimicrobial activity of *Capparis spinosa* as its usages in traditional medicine. Herba Pol. 60, 39–48. 10.2478/hepo-2014-0004

[B113] Maire R. (1965). Flore de l'Afrique du Nord. Encycl. Biol. 67, 256–302.

[B114] Mansour B. R. Jilani H. B. I. Bouaziz M. Gargouri B. Elloumi N. Attia H.. (2016). Phenolic contents and antioxidant activity of ethanolic extract of *Capparis spinosa*. Cytotechnology 68, 135–142. 10.1007/s10616-014-9764-6 25377263 PMC4698273

[B115] Maresca M. Micheli L. Mannelli L. D. C. Tenci B. Innocenti M. Khatib M.. (2016). Acute effect of *Capparis spinosa* root extracts on rat articular pain. J. Ethnopharmacol. 193, 456–465. 10.1016/j.jep.2016.09.032 27647009

[B116] Matsuyama K. Villareal M. O. Omri A. E. Han J. Kchouk M. E. Isoda H. (2009). Effect of Tunisian *Capparis spinosa* L. extract on melanogenesis in B16 murine melanoma cells. J. Nat. Med. 63, 468–472. 10.1007/s11418-009-0355-3 19685105

[B117] Matthäus B. Özcan M. (2005). Glucosinolates and fatty acid, sterol, and tocopherol composition of seed oils from *Capparis spinosa* var. spinosa and Capparis ovata Desf. var. canescens (Coss.) *Heywood*. J. Agric. Food. Chem. 53, 7136–7141. 10.1021/jf051019u 16131121

[B118] Matthäus B. Ozcan M. (2002). Glucosinolate composition of young shoots and flower buds of capers (*Capparis* Species) growing wild in Turkey. J. Agric. Food. Chem. 50, 7323–7325. 10.1021/jf020530+ 12452652

[B119] Mithen F. R. Dekker M. Verkerk R. Rabot S. Johnson I. T. (2000). The nutritional significance, biosynthesis and bioavailability of glucosinolates in human food. J. Sci. Food Agric. 80, 967–984. 10.1002/(SICI)1097-0010(20000515)80:7<967::AID-JSFA597>3.0.CO;2-V

[B120] Mollica A. Zengin G. Locatelli M. Stefanucci A. Mocan A. Macedonio G. (2017). Anti-diabetic and anti-hyperlipidemic properties of *Capparis spinosa* L.: *in vivo* and *in vitro* evaluation of its nutraceutical potential. J. Funct. Foods. 35, 32–42. 10.1016/j.jff.2017.05.001

[B121] Moubasher H. Abd El-Ghani M. M. Kamel W. Mansi M. El-Bous M. (2011). Taxonomic considerations among and within some Egyptian taxa of *Capparis* and related genera (Capparaceae) as revealed by RAPD fingerprinting. Collect Bot. 3, 29–35. 10.3989/collectbot.2011.v30.003

[B122] Moufid A. Farid O. Eddouks M. (2015). Pharmacological Properties of *Capparis spinosa* Linn. Int. J. Diabetol. Vasc. Dis. Res. 3, 99–104.

[B123] Mousavi S. H. Housseini A. Bakhtiari E. Rakhshandeh H. (2016). *Capparis spinosa* reduced Doxorubicin-induced cardiotoxicity in Cardiomyoblast cells. Avcenna J. Phytomed. 6, 488–494. PMC5052410 27761417

[B124] Mouterde P. (1968). Nouvelle flore du Liban et de la Syrie. Beirut: Imprimerie Catholique.

[B125] Muhaidat R. Al-Qudah A. M. Al-Shayeb A. Jacob H. J. Al-Jaber H. Hussein E. (2013). Chemical profile and antibacterial activity of crude fractions and essential oils of *Capparis ovata* Desf. And *Capparis spinosa* L. (Capparaceae). Int. J. Integ. Biol. 14, 39–47.

[B126] Murzin V. S. (1986). Diurnal Lepidoptera (Rhopalocera) of the Badkhyzskii Reserve (Turkmen, SSR). Trudy Vsesoyuznogo Entomologisheskogo Obshchestva, Akademiya Nauk SSSR. 6, 125–130.

[B127] Musallam I. Duwayri M. Shibli R. (2010). Micropropagation of caper (*Capparis spinosa* L.) from wild plants. Funct Plant Sci Biotechnol. 5, 17–21.

[B128] Nabavi S. F. Maggi F. Daglia M. Habtemariam S. Rastrelli L. Nabavi S. M. (2016). Pharmacological effects of *Capparis spinosa* L. Phytother. Res. 30, 1733–1744. 10.1002/ptr.5684 27406313

[B129] Neyisci T. (1987). A study on the slow burning plant species suitable for controlling forest fires' (in Turkish, summary in English). Turk. J. Agric. For. 11, 595–604.

[B130] Nosrati H. Feizi H. A. M. Mazinani M. Haghighi R. A. (2012). Effect of population size on genetic variation levels in *Capparis spinosa* (Capparaceae) detected by RAPDs. EurAsia J. BioSci. 6, 70–75. 10.5053/ejobios.2012.6.0.8

[B131] Ohama N. Sato H. Shinozaki K. Yamaguchi-Shinozaki K. (2017). Transcriptional regulatory network of plant heat stress response. Trends Plant Sci. 22, 53–65. 10.1016/j.tplants.2016.08.015 27666516

[B132] Oppenheimer H. R. (1960). Adaptation to drought; xerphytism, in UNESCO, Plant-Water Relationships in Arid and Semi-Arid Conditions, ed Reviews of Research (New York, NY: United Nations), 105–138.

[B133] Ortiz R. Braun H. J. Crossa J. Crouch J. H. Davenport G. Dixon J. (2008). Wheat genetic resources enhancement by the International Maize and Wheat Improvement Center (CIMMYT). Genet. Resour. Crop Evol. 55, 1095–1140. 10.1007/s10722-008-9372-4

[B134] Orphanides G. M. (1975). Biology of the carob midge complex, *Asphondylia* spp. (Diptera, Cecidomyiidae) in Cyprus. Bull. Entomol. Res. 65, 381–390. 10.1017/S0007485300006052

[B135] Orphanos P. I. (1983). Germination of caper (*Capparis spinosa* L.) seeds. J. Hortic. Sci. 58, 267–270. 10.1080/00221589.1983.11515119

[B136] Ozbek O. Kara A. (2013). Genetic variation in natural populations of *Capparis* from Turkey, as revealed by RAPD analysis. Plant Syst. Evol. 299, 1911–1933. 10.1007/s00606-013-0848-0

[B137] Özkahraman I. (1997). Caper. Forest Ministry, Various publications Series Nos 2. Ankara: AGM publications.

[B138] Pachauri R. K. Allen M. R. Barros V. R. Broome J. Cramer W. Christ R. (2014). Climate change 2014: synthesis report, in Contribution of Working Groups I, II and III to the Fifth Assessment Report of the Intergovernmental Panel on Climate Change, ed Pachauri R. Meyer L. (Geneva: IPCC), 151.

[B139] Panico A. M. Cardile V. Garufia F. Pugliaa C. Boninaa F. Ronsisvalle G. (2005). Protective effect of *Capparis spinosa* on chondrocytes. Life Sci. 77, 2479–2488. 10.1016/j.lfs.2004.12.051 15946691

[B140] Pascual B. San Bautista A. Ferreros N. Lopez-Galarza S. Maroto J. V. (2003). Analysis of germination of caper seeds as influenced by the position of fruit on the mother plant, fruit maturation stage and fruit weight. J. Hortic. Sci. Biotech 78, 73–78.

[B141] Peri E. Lo Bue P. Federico R. Ammavuta G. Spatafora F. Colazza S. (2006). *Asphondylia gennadii* (Marchal) fitofago dannoso al cappero nelle isole minori della Sicilia (Diptera: Cecidomyiidae). Inf. Fitopatol. 56, 26–30.

[B142] Pilone N. (1990). Effetti dell'IBA sulla radicazione delle talee di *Capparis spinosa* in cassone riscaldato. Inf. Agrar. 46, 81–82.

[B143] Pittaway A. R. (1979). The butterflies and hawk-moths of Eastern Saudi Arabia. Proc. Br. Entomol. Nat. Hist. Soc. 12, 90–101.

[B144] Post G. E. (1932). Flora of Syria, Palestine and Sinai. Beirut: American University of Beirut.

[B145] Prasad P. V. V. Staggenborg S. A. Ristic Z. (2008). Impacts of drought and/or heat stress on physiological, developmental, growth, and yield processes of crop plants, in Response of Crops to Limited Water: Understanding and Modeling Water Stress Effects on Plant Growth Processes, ed Ahuja L. R. Reddy V. R. Saseendran S. A. Yu Q. (Madison, WI: American Society of Agronomy, Crop Science Society of America, Soil Science Society of America), 301–355.

[B146] Psaras G. K. Sofroniou I. (1999). Wood anatomy of *Capparis spinosa* from an ecological perspective. IAWA J. 20, 419–429. 10.1163/22941932-90001567

[B147] Rahmani R. Mahmoodi M. Karimi M. Hoseini F. Heydari R. Salehi M. (2013). Effect of hydroalcoholic extract of *Capparis spinosa* fruit on blood sugar and lipid profile of diabetic and normal rats. Zahedan J. Res. Med. Sci. 15, 34–38.

[B148] Rahnavard R. Razavi N. (2016). A review on the medical effects of *Capparis spinosa* L. Adv. Herb. Med. 2, 44–53.

[B149] Rangarajan A. V. Mahadewan N. R. (1975). Incidence of gallmidge, Asphondylia capsici Barnes on chilli in Tamil. Indian J. Entomol. 36, 66–67.

[B150] Rapisarda C. (1985). Presenza in Italia di Aleurolobus niloticus Priesner & Hosny, nuovo parassita delle piante di cappero (Homoptera: Aleirodidae). Bollettino di Zoologia agraria e Bachicoltura. 18, 75–86.

[B151] Ray D. K. Gerber J. S. MacDonald G. K. West P. C. (2015). Climate variation explains a third of global crop yield variability. Nat. Commun. 6:5989. 10.1038/ncomms6989 25609225 PMC4354156

[B152] Renfrew J. M. (1973). Palaeoethnobotany. The Prehistoric Food Plants of the Near East and Europe. New York, NY: Columbia University Press.

[B153] Rhizopoulou S. (1990). Physiological responses of *Capparis spinosa* L. to drought. J. Plant Physiol. 136, 341–348. 10.1016/S0176-1617(11)80060-X

[B154] Rhizopoulou S. Heberlein K. Kassianou A. (1997). Field water relations of *Capparis spinosa* L. J. Arid. Environ. 36, 237–248. 10.1006/jare.1996.0207

[B155] Rhizopoulou S. Loannidi E. Alexandredes N. Argiropoulos A. (2006). A study of functional and structural traits of the nocturnal flowers of *Capparis spinosa* L. J. Arid. Environ. 66, 635–647. 10.1016/j.jaridenv.2005.12.009

[B156] Rhizopoulou S. Psaras G. K. (2003). Development and structure of drought-tolerant leaves of the Mediterranean shrub *Capparis spinosa* L. Ann. Bot. 92, 377–383. 10.1093/aob/mcg149 12853284 PMC4257511

[B157] Rivera D. Alcaraz F. Inocencio C. Obón C. Carreño E. (1999). Taxonomic study of cultivated Capparis sect. Capparis in the western Mediterranean, in Taxonomy of Cultivated Plants, ed Andrew S. Leslie A. C. Alexander C. (England: Royal Botanic Gardens), 451–455.

[B158] Rivera D. Inocencio C. Obon C. Alcaraz F. (2003). Review of food and medicinal uses of *Capparis* L. subgenus Capparis (Capparidaceae). Econ. Bot. 57, 515–534. 10.1663/0013-0001(2003)057[0515:ROFAMU]2.0.CO;2

[B159] Rivera D. Inocencio C. Obon C. Carreno E. Reales A. Alcaraz F. (2002). Archaeobotany of Capers (Capparis) (Capparaceae). Veg. Hist. Archaeobot. 11, 295–314. 10.1007/s003340200042

[B160] Rodriguez R. Rey M. Cuozzo L. Ancora G. (1990). *In vitro* propagation of caper (*Capparis spinosa* L.). *In Vitro* Cell. Dev. Biol. 26, 531–536. 10.1007/BF02624097

[B161] Romeo V. Ziino M. Giuffrida D. Condurso C. Verzera A. (2007). Flavor profile of Capers (*Capparis spinosa* L.) from the Eolian Archipelago by HS-SPME/GC-MS. Food Chem. 101, 1272–1278. 10.1016/j.foodchem.2005.12.029

[B162] Rotondi A. Rossi F. Asunits C. Cesaraccio C. (2003). Leaf xeromorphic adapatations of some plants of coastal Mediterranean ecosystem. J. Meditternean Ecol. 3, 25–35.

[B163] Saadaoui E. Guetat A. Tlili N. El Gazzah M. Khaldi A. (2011). Subspecific variability of Tunisian wild populations of *Capparis spinosa* L. J. Med. Plants Res. 5, 4339–4348.

[B164] Saadaoui E. Khaldi A. Khouja M. L. El-Gazzah M. (2007). Etude de la variabilité morphologique du câprier (*Capparis* spp.) en Tunisie. Revue des Régions Aride. 2, 523–527.

[B165] Sakcali M. Bahadir H. Ozturk M. (2008). Eco-physiology of *Capparis spinosa* L. A plant suitable for combating desertification. Pak. J. Bot. 40, 1481–1486.

[B166] Saifi N. Ibijbijen J. Echchghadda G. (2011). Genetic diversity of Caper plant (*Capparis* sp.) from Morocco. J. Food Agric. Environ. 9, 299–304.

[B167] Salem A. Zemni H. Ghorbel A. (2001). Propagation of caper (*Capparis spinosa* L.) by herbaceous cuttings and *in vitro* culture. Agric. Med. 31, 42–48.

[B168] Selfayan M. Namjooyan F. (2016). Inhibitory *Effect of Capparis spinosa* Extract on Pancreatic Alpha-Amylase Activity. Zahedan J. Res. Med. Sci. 18:e6450 10.17795/zjrms-6450

[B169] Sharaf M. El-Ansari A. M. Saleh N. A. M. (1997). Flavonoids of four *Cleome* and three *Capparis* species. Biochem. Syst. Ecol. 25, 161–166 10.1016/S0305-1978(96)00099-3

[B170] Sharaf M. El-Ansari A. M. Saleh N. A. (2000). Quercetin triglycoside from *Capparis spinosa*. Fitoterapia. 71, 46–49. 10.1016/S0367-326X(99)00116-1 11449469

[B171] Sheikh K. H. (1976). Variations in leaf hydration and stomatal openings of some maquis in response to changes in some environmental factors, in Proceedings of the Third Mediterranean Plant Physiology Meeting, ed Vardar Y. Sheikh K. H. Ozturk M. (Izmir: Ege University Press), 24–36.

[B172] Sher H. Al-Mutayri K. Mansoor M. (2012). Study on the ethnopharmaceutical values and traditional uses of *Capparis spinosa* L. Afr. J. Pharm. Pharmamcol. 6, 1255–1259.

[B173] Sher H. Alyemeni M. (2010). Ethnobotanical and pharmaceutical evaluation of *Capparis spinosa* L, validity of local folk and Unani system of medicine. J. Med. Plants Res. 4, 1751–1756.

[B174] Silvestre G. A. Silvio F. Mirko S. Ignazio F. Giuseppe G. Francesco C. (2014). Hybridization in *Capparis spinosa* L.: molecular and morphological evidence from a Mediterranean island complex. Flora 209, 733–741. 10.1016/j.flora.2014.09.002

[B175] Singh R. P. Bahar N. Chand P. (1992). Autecology of *Capparis spinosa* Linn. in cold desert of Spiti Valley in Himachal Pradesh. Ann. Arid. Zone. 31, 291–293.

[B176] Solowey E. (2010) “Arboreal pastures” in Growing Bread on Trees: The Case for Perennial Agriculture. Miami, FL: Acco, Israel/Biblio Books International.

[B177] Sozzi O. G. (2001). Caper bush: botany and horticulture. Hortic. Rev. 27, 125–188. 10.1002/9780470650813.ch4

[B178] Sozzi O. G. Chiesa A. (1995). Improvement of caper (*Capparis spinosa* L.) seed germination by breaking seed coat-induced dormancy. Sci. Hortic. 62, 255–261. 10.1016/0304-4238(95)00779-S

[B179] Sozzi O. G. Vicente A. R. (2006). Capers and caperberries, in Handbook of Herbs and Spices, ed Peter K. V. (Boca Raton, FL: Woodhead Publishing Limited; CRC Press), 230–256.

[B180] Stefanou M. Manetas Y. (1997). The effect of season, exposure, enhanced UV-B radiation, and water stress on leaf epicuticular and internal UV-B absorbing capacity of *Cistus creticus*: a Mediterranean field study. J. Exp. Bot. 48, 1977–1985. 10.1093/jxb/48.11.1977

[B181] Thiry A. A. Dulanto P. N. C. Reynolds M. P. Davies W. J. (2016). How can we improve crop genotypes to increase stress resilience and productivity in a future climate? A new crop screening method based on productivity and resistance to abiotic stress. J. Exp. Bot. 67, 5593–5603. 10.1093/jxb/erw330 27677299 PMC5066489

[B182] Tlili N. El-Fallah W. Saadadoui E. Khaldi A. H. Triki S. Nasri N. (2011a). The caper (*Capparis* L.): ethnopharmacology, phytochemical and pharmacological properties. Fitoterapia 82, 93–101. 10.1016/j.fitote.2010.09.006 20851750

[B183] Tlili N. El Guizani T. Nasri N. Khaldi A. Triki S. (2011b). Protein, lipid, aliphatic and triterpenic alcohol content of Caper seeds “*Capparis spinosa*”. J. Am. Oil Chem. Soc. 88, 265–270. 10.1007/s11746-010-1662-2

[B184] Tlili N. Feriani A. Saadoui E. Nasri N. Khaldi A. (2017). *Capparis spinosa* leaves extract: source of bioantioxidants with nephroprotective and hepatoprotective effects. Biomed. Pharmacother. 87, 171–179. 10.1016/j.biopha.2016.12.052 28056421

[B185] Tlili N. Khaldi A. Triki S. Munné-Bosh S. (2010). Phenolic compounds and vitamin antioxidants of Caper (*Capparis spinosa*). Plant Foods Hum. Nutr. 65, 260–265. 10.1007/s11130-010-0180-6 20668946

[B186] Tomassoli L. Zaccaria A. Barba M. (2005). *Capparis spinosa*, a new host of *cucumber mosaic virus* in Italy. Plant Pathol. J. 54:263 10.1111/j.1365-3059.2005.01125.x

[B187] Trombetta D. Occhiuto F. Perri D. Puglia C. Santagati N. A. Pasquale A. D.. (2005). Antiallergic and antihistaminic effect of two extracts of *Capparis spinosa* L. flowering buds. Phytother. Res. 19, 29–33. 10.1002/ptr.1591 15799005

[B188] UNEP/ROWA (United Nations Environment Programme, Regional Office for West Asia) (2015). Climate Change in the Arab Region. Regional Coordination Mechanism. Issues Brief for the Arab Sustainable Development Report. Manama.

[B189] Vahid H. Rakhshandeh H. Ghorbani A. (2017). Antidiabetic properties of *Capparis spinosa* L. and its components. Biomed. Pharmacother. 92, 293–302. 10.1016/j.biopha.2017.05.082 28551550

[B190] Van Z. W. Bakker-Heeres J. A. H. (1988). Archaeobotanical studies in the Levant. 4. Bronze Age sites on the North Syrian Euprates. Palaeohistoria 27, 247–316.

[B191] Vardar Y. Ahmed M. (1972). Relative transpiration of the old and young leaves of some macchina elements. Phyton 14, 251–262.

[B192] Velikova V. Pinelli P. Pasqualini S. Reale L. Ferranti F. Loreto F. (2005). Isoprene decreases the concentration of nitric oxide in leaves exposed to elevated ozone. New Phytol. 166, 419–426. 10.1111/j.1469-8137.2005.01409.x 15819906

[B193] Vollenweider P. Günthardt-Goerg M. S. (2005). Diagnosis of abiotic and biotic stress factors using the visible symptoms in foliage. Environ. Pollut. 137, 455–465. 10.1016/j.envpol.2005.01.032 16005758

[B194] Wahid A. (2007). Physiological implications of metabolites biosynthesis for net assimilation and heat- stress tolerance of sugarcane (*Saccaharum officinarum)* sprouts. J. Plant Res. 120, 219–228. 10.1007/s10265-006-0040-5 17024517

[B195] Wang Q. Zhang M. L. Yin L. K. (2016). Phylogeographic structure of a tethyan relict *Capparis spinosa* (Capparaceae) traces pleistocene geologic and climatic changes in the western Himalayas, Tianshan mountains, and adjacent desert regions. Biomed. Res. Int. 2016:13. 10.1155/2016/5792708 27314028 PMC4903145

[B196] Wealth of India (1992). A Dictionary of Indian Raw Material and Industrial Products. New Delhi: CSIR.

[B197] Willis J. C. (1988). A Dictionary of the Flowering Plants and Ferns. Cambridge: Cambridge Cambrigde University Press.

[B198] Xu Z. Jiang Y. Zhou G. (2015). Response and adaptation of photosynthesis, *respiration*, and antioxidant systems to elevated CO_2_ with environmental stress in plants. Front. Plant Sci. 6:701. 10.3389/fpls.2015.00701 26442017 PMC4564695

[B199] Yamori W. Hikosaka K. Way D. A. (2014). Temperature response of photosynthesis in C3, C4, and CAM plants: temperature acclimation and temperature adaptation. Photosynth. Res. 119, 101–117. 10.1007/s11120-013-9874-6 23801171

[B200] Yang T. Wang C. Chou G. X. Wu T. Cheng X. M. Wang Z. T. (2010b). New alkaloids from *Capparis spinosa*: structure and X-ray crystallographic analysis. Food Chem. 123, 705–710. 10.1016/j.foodchem.2010.05.039

[B201] Yang T. Wang C. Liu H. Chou G. Cheng X. Wang Z. (2010a). A new antioxidant compound from *Capparis spinosa*. Pharm. Biol. 48, 589–594. 10.3109/13880200903214231 20645804

[B202] Yu L. Yang J. Wang X. Jiang B. Sun Y. Ji Y. (2017). Antioxidant and antitumor activities of *Capparis spinosa* L. and the related mechanisms. Oncol. Rep. 37, 357–367. 10.3892/or.2016.5249 27878299

[B203] Zhang S. Hu D. B. He J. B. Guan K. Y. Zhu H. J. (2014). A novel tetrahydroquinoline acid and a new racemic benzofuranone from *Capparis spinosa* L., a case study of absolute configuration determination using quantum methods. Tetrahedron 70, 869–873. 10.1016/j.tet.2013.12.024

[B204] Zhou H. Jian R. Kang J. Huang X. Li Y. Zhuang C.. (2010). Anti-inflammatory effects of Caper (*Capparis spinosa* L.) fruit aqueous extract and the isolation of main phytochemicals. J. Agric. Food Chem. 58, 12717–12721. 10.1021/jf1034114 21105652

[B205] Zhou X. Liu Y. (2015). Hybridization by grafting: a new perspective? HortScience 50, 520–521.

[B206] Ziroyan A. N. (1980). Seed productivity and renewal of some semidesert plant species on the large southern slope of Mount Aragats, Armenian SSR, USSR' (in Russian). Biol. Zh. Arm. 33, 91–94.

[B207] Zohary M. (1960). The species of *Capparis* in the Mediterranean and the Near Eastern countries. Bull. Res. Counc. Isr. 8D, 49–64.

[B208] Zuo W. Ma M. Ma Z. Gao R. Guo Y. Jiang W. (2012). Study of photosynthetic physiological characteristics of desert plant *Capparis spinosa* L. J. Shihezi Univ. 3:006.

